# Advances in secondary prevention mechanisms of macrovascular complications in type 2 diabetes mellitus patients: a comprehensive review

**DOI:** 10.1186/s40001-024-01739-1

**Published:** 2024-03-04

**Authors:** Huifang Guan, Jiaxing Tian, Ying Wang, Ping Niu, Yuxin Zhang, Yanjiao Zhang, Xinyi Fang, Runyu Miao, Ruiyang Yin, Xiaolin Tong

**Affiliations:** 1 College of Traditional Chinese Medicine, Changchun University of Chinese Medicine, Changchun, 130117 China; 2 Institute of Metabolic Diseases, Guang’anmen Hospital, China Academy of Chinese Medical Sciences, Beijing, 100053 China; 3 Rehabilitation Department, The Affiliated Hospital of Changchun University of Chinese Medicine, Changchun, 130021 China; 4 Graduate College, Beijing University of Chinese Medicine, Beijing, China

**Keywords:** Type 2 diabetes mellitus (T2DM), Macrovascular complications, Secondary prevention, Protective mechanisms, Experimental techniques and methodologies

## Abstract

Type 2 diabetes mellitus (T2DM) poses a significant global health burden. This is particularly due to its macrovascular complications, such as coronary artery disease, peripheral vascular disease, and cerebrovascular disease, which have emerged as leading contributors to morbidity and mortality. This review comprehensively explores the pathophysiological mechanisms underlying these complications, protective strategies, and both existing and emerging secondary preventive measures. Furthermore, we delve into the applications of experimental models and methodologies in foundational research while also highlighting current research limitations and future directions. Specifically, we focus on the literature published post-2020 concerning the secondary prevention of macrovascular complications in patients with T2DM by conducting a targeted review of studies supported by robust evidence to offer a holistic perspective.

## Introduction

Type 2 diabetes mellitus (T2DM) has become a global health crisis, with projections indicating that more than 693 million adults will be affected by the year 2045 [1]. While hyperglycemia is a hallmark of T2DM, the primary contributor to increased mortality risk in these patients is macrovascular complications induced by atherosclerosis, such as coronary artery disease (CAD), cerebrovascular disease, and peripheral artery disease (PAD) [1]. Importantly, atherosclerosis is not limited to just one specific vascular bed. Research indicates that many T2DM patients have atherosclerotic changes in not just one but multiple vascular beds. This multisite vascular involvement substantially increases the risk of macrovascular complications, particularly in the context of T2DM [2]. Thus, targeted secondary prevention measures for this high-risk population are imperative. Concurrently, the public health burden of T2DM is causing a precipitous decline in patient quality of life, significant economic and psychological strains on families and unprecedented challenges to healthcare systems [3]. Regrettably, despite advances in various medical fields, current therapeutic strategies for addressing T2DM-related macrovascular complications are inadequate. This highlights the urgency and importance of effective secondary prevention for diagnosed T2DM patients.

Given this background, the objective of this review was to systematically and comprehensively explore secondary prevention mechanisms for macrovascular complications in T2DM patients. We initially delve into the underlying pathophysiological mechanisms of this disease, such as dysregulation of glucose metabolism, chronic inflammation, and alterations in hemorheology, and discuss how these collectively contribute to the development of macrovascular complications in T2DM patients. Subsequently, we focus on various preventive strategies, ranging from biomarker monitoring and pharmacological interventions to lifestyle modifications aimed at mitigating the risk of macrovascular complications. Additionally, we examine the experimental models and techniques employed in the research on macrovascular complications in T2DM patients, particularly those leveraging bioengineering and medical imaging modalities.

In summary, this review not only offers a comprehensive overview of secondary prevention of macrovascular complications in T2DM patients but also identifies existing gaps in the research and future research directions. Given the voluminous nature of the related literature, particular attention is given to studies published after 2020, selectively discussing those with compelling evidence. Despite significant advances in this field in recent years, numerous challenges remain to be addressed, and it is our hope that this review will provide valuable guidance in navigating these complexities.

## Pathophysiological mechanisms of macrovascular complications in diabetes

**Fig. 1:**
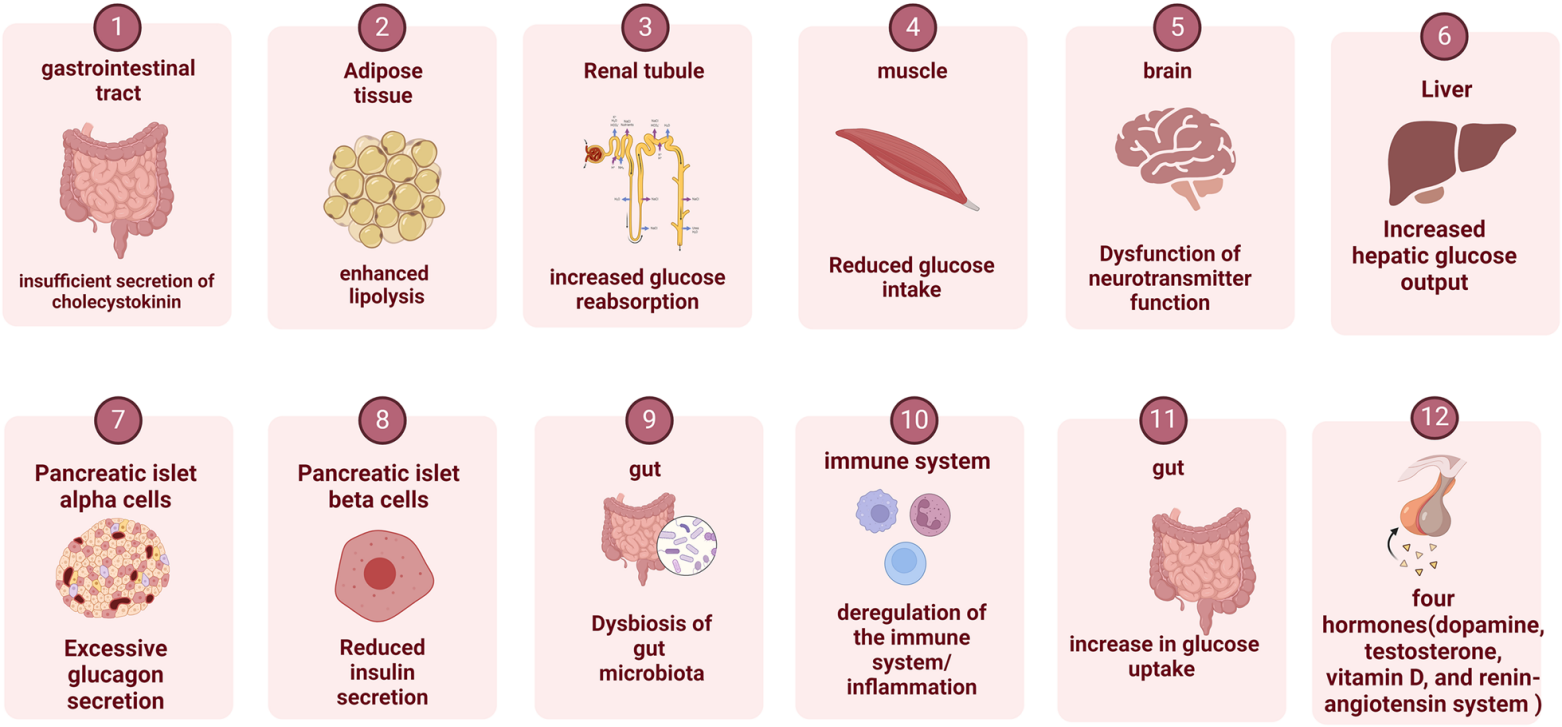
The dirty dozen of diabetes

**Fig. 2:**
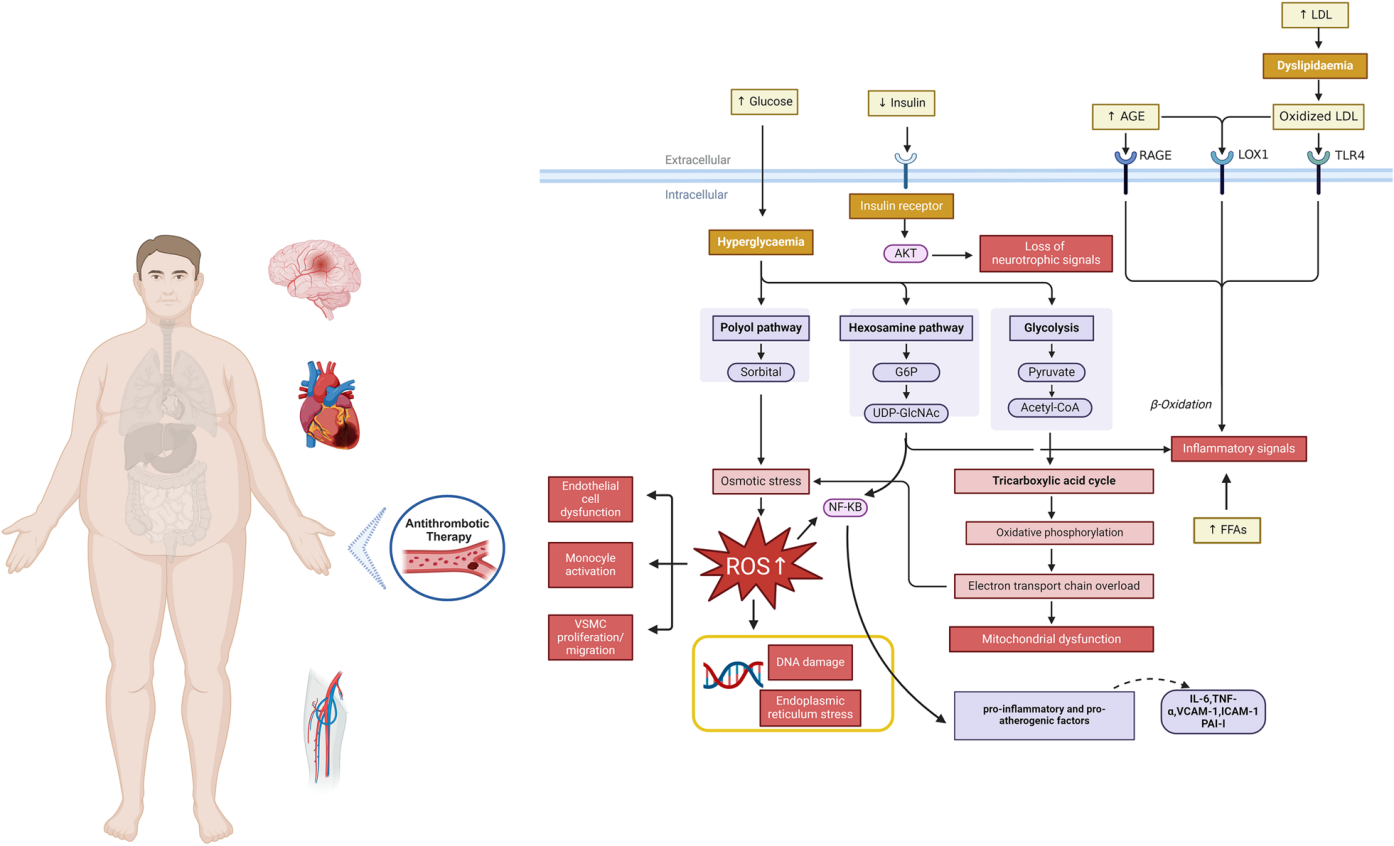
Pathophysiological mechanisms of diabetes and its complications

Macrovascular complications in diabetes involve a myriad of complex pathophysiological processes, at least 12 pathophysiological abnormalities, also known as the "the dirty dozen of diabetes" are recognized, and additional unknown mechanisms of disease are under investigation [4] (Fig. 1). Currently, research primarily considers its pathophysiology to be driven by persistent hyperglycemia, insulin resistance, and dyslipidemia (Fig. 2). Additionally, the cellular response to toxic metabolites from glucose metabolism exacerbates the formation of atherosclerotic lesions and plaques, accelerating endothelial dysfunction and oxidative stress [4, 5]. Long-term diabetic states, inconsistent glycemic control, factors related to genetic and epigenetic predispositions, and underlying comorbidities collectively increase the risk of macrovascular complications in patients with T2DM [5, 6]. Notably, comorbid conditions, such as hypertension and obesity, further modulate intracellular glucose concentrations, triggering cascading biochemical reactions that exacerbate vascular deterioration [5].

### Basic vascular structure and function

The vascular system comprises an intricate assembly of key cellular constituents, such as endothelial cells (ECs) and smooth muscle cells, that collectively ensure the integrity and functionality of blood vessels [7]. In particular, ECs are pivotal regulators of vascular health across multiple organs and are tasked with maintaining vascular tone, enabling angiogenesis, and executing antioxidant defenses [8]. These cells exhibit significant functional heterogeneity, which is essential not only for meeting the physiological demands of the organs they serve but also for responding to mechanical forces such as shear stress [9, 10]. Shear stress, the frictional force exerted by blood flow, is a fundamental determinant of EC function [10]. ECs are exquisitely sensitive to the shear stress levels that vary across the vascular tree—being higher in straight segments and altered at arterial bifurcations and bends. This mechanical force is instrumental in shaping EC behavior, influencing gene expression, and cellular adaptation in response to the hemodynamic environment [10]. In regions of uniform high shear stress, ECs maintain a quiescent, anti-inflammatory state that is protective against vascular diseases. Conversely, at sites of disturbed flow, ECs may undergo functional changes that predispose them to pathology, setting the stage for disease processes such as atherosclerosis [10].

The primary role of the vascular system in facilitating the efficient transport of oxygen, nutrients, and bioactive molecules as well as the clearance of metabolic waste is largely contingent upon the heterogeneity and adaptability of ECs [7]. In the setting of T2DM, disruptions in glucose metabolism may compromise the delicate equilibrium of EC function, leading to impaired angiogenesis, endothelial-to-mesenchymal transition, and a cascade of vascular dysfunctions that manifest as thickening, hardening, and functional decline of the vascular wall [8, 9]. These pathological alterations, exacerbated by the inherent heterogeneity of ECs, act in concert to precipitate macrovascular complications, underscoring the imperative for therapeutic strategies that address this cellular diversity [7]. In the context of T2DM, a chronic hyperglycemic state perturbs the nuanced balance of EC functions, amplifying their heterogeneity and impacting their physiological responsiveness. The complex signaling networks involving neuropilins, vascular endothelial growth factor/vascular endothelial growth factor receptor 2, NOTCH-Dll4, and Kruppel-like factors, which orchestrate arteriovenous specification and shear stress responses, become dysregulated [9]. Metabolic alterations and endothelial-to-mesenchymal transition have emerged as novel hallmarks of this dysfunction, contributing to compromised angiogenesis and vascular regenerative capacity across a spectrum of vascular diseases, including atherosclerosis, hypertension, and diabetes [8, 9]. This dysregulation is critical to the pathophysiological understanding of macrovascular complications in diabetes, as it influences EC behavior, promotes endothelial dysfunction, and contributes to the vascular pathological characteristics of T2DM.

### Pathophysiological changes in type 2 diabetes mellitus patients

#### Evolution of atherosclerosis

In patients with T2DM, macrovascular complications are primarily characterized by the progression of atherosclerosis, manifesting as foam cell formation in macrophages, EC damage, and smooth muscle cell injury [7]. Pathogenesis is initiated by the deposition of lipoproteins on arterial walls, leading to foam cell accumulation within the subendothelial space. Here, low-density lipoprotein (LDL) particles undergo oxidation, culminating in structural alterations in vascular architecture [5]. Macrophages are central to atherogenesis, mediating lipid uptake and fostering vascular inflammation, thereby augmenting lesion complexity via their phenotypic versatility [11]. Additionally, the dysregulation of macrophage function, modulated by genetic and environmental determinants, markedly accelerates the progression of atherosclerosis [12]. Elevated extracellular glucose concentrations have been shown to induce proinflammatory gene expression and atherogenic traits in macrophages through glycolysis-dependent pathways [13]. The lipid molecule 25-hydroxycholesterol (25-HC) is particularly significant because it accumulates in atherosclerotic lesions and hastens disease progression by enhancing inflammatory responses and impeding smooth muscle cell repair mechanisms [14]. In addition to macrophages, ECs and smooth muscle cells (SMCs) also play significant roles in various stages of atherosclerosis development. ECs regulate the ingress of blood components, including lipids and cells, into the arterial wall, thereby influencing key processes, such as inflammation, SMC proliferation and migration, vascular tone, and coagulation [10, 15]. SMCs display remarkable adaptability, dynamically adjusting their phenotype in response to environmental changes [11]. Recent lineage-tracing studies have indicated that SMCs not only contribute to the formation of protective fibrous caps and extracellular matrix synthesis but also actively participate in cytokine production, lipid accumulation, phagocytosis of apoptotic cells, and calcification [11]. Recent studies have underscored the exacerbating effect of diabetes on coronary artery pathology, particularly the dysregulation of signaling proteins such as S-nitrosylated guanine nucleotide-binding protein G(i) alpha-2, which is implicated in the amplification of endothelial inflammation—a key factor in the progression of atherosclerosis in T2DM patients [16].

Although atherosclerosis is linked to systemic risk factors such as hypercholesterolemia and advanced age, it predominantly occurs near arterial branches and bends [10]. The chronic hyperglycemic state of T2DM exacerbates the impact of disturbed shear stress on vascular pathology. At arterial branches and bends, where blood flow is inherently complex, low or oscillatory shear stress promotes the pathogenesis of atherosclerosis by inducing vascular inflammation and dysfunction [10]. These areas are particularly susceptible to the development of atherosclerotic lesions, which are characterized by EC proliferation, apoptosis, and increased permeability to cholesterol-rich lipoproteins [17]. Conversely, laminar flow exerts a protective effect.

In T2DM, the pathogenesis of atherosclerosis is not merely a localized vascular phenomenon but also a reflection of a panvascular process, where systemic inflammation is intricately associated with plaque instability and rupture [18]. This broader view of atherosclerosis as a systemic inflammatory condition is supported by evidence of uniformly elevated macrophage density, indicative of inflammation, at sites of both ruptured and intact lesions [18]. Such insights have propelled a paradigm shift from a singular focus on vulnerable plaques to a recognition of the entire coronary tree's susceptibility to inflammatory processes. This systemic vulnerability is particularly pronounced in T2DM, where metabolic disturbances amplify the deleterious effects of shear stress on the vasculature, thereby accelerating the progression of atherosclerotic lesions [10]. Advanced imaging modalities, including optical coherence tomography, have been instrumental in elucidating the role of hemodynamic forces in plaque vulnerability, reinforcing the importance of thin-cap fibroatheromas and related biomarkers in the early detection and targeted management of macrovascular complications in T2DM patients [19].

#### Interplay of insulin resistance, hyperglycemia, and β-cell dysfunction

T2DM is characterized by chronic hyperglycemia, a consequence of insulin resistance coupled with the progressive loss of β-cell function. These central pathophysiological features of T2DM have widespread deleterious effects on the cardiovascular system [20, 21]. Insulin resistance, in particular, is a critical factor that not only underlies the persistent hyperglycemia characteristic of T2DM but also contributes to macrovascular complications, independent of the glycemic status [22]. It typically manifests during the prediabetic stage and is often accompanied by hyperinsulinemia, which is a result of compensatory overproduction of insulin by β-cells [23]. Furthermore, insulin resistance is associated with a range of metabolic abnormalities, such as obesity, hyperinsulinemia, and hyperlipidemia. Adipose tissue plays a crucial role in this process by secreting lipids and other circulating factors that promote insulin resistance in other organs [24]. Recent studies have revealed that exogenous fibroblast growth factor 1 mitigates diabetes through its acute inhibition of hepatic glucose production and lipolysis via adipose fibroblast growth factor receptor 1 [25, 26].

Persistent insulin resistance in T2DM patients paves the way for sustained hyperglycemia, which is the precursor to a host of detrimental vascular effects. This elevated glucose milieu facilitates the nonenzymatic glycation of proteins and lipids, leading to the generation of advanced glycation end products (AGEs) [27]. These AGEs, through their interaction with receptor for advanced glycation end product (RAGE), incite inflammatory responses and oxidative stress, mechanisms that are central to the pathogenesis of both micro- and macrovascular complications, including atherosclerosis [27]. Furthermore, the glycosylation of specific proteins, such as fibrinogen, which is mediated by chronic hyperglycemia, is recognized as a pivotal factor in the development of these vascular complications, underscoring the intricate link between metabolic dysregulation and vascular pathology in diabetes mellitus [28]. Moreover, fluctuations in both short-term and long-term glycemic levels have been correlated with macrovascular complications in diabetes patients [29]. This is particularly evident in patients with transient intermittent hyperglycemia, even among those who generally maintain good glycemic control [29]. Hyperglycemia exerts a profound influence on the immune system by inducing trained immunity in hematopoietic stem cells and macrophages, leading to persistent inflammatory responses mediated by the activation of RUNX1, a transcription factor pivotal in inflammatory and metabolic pathways [13]. This phenomenon of hyperglycemia-induced trained immunity may elucidate the limited efficacy of traditional glucose-lowering therapies in diminishing the risk of atherosclerotic vascular disease in diabetic patients [13]. Additionally, studies have identified oxidative modifications in key components of the antigen processing and presentation mechanisms of major histocompatibility complex class II molecules under high-glucose conditions [30]. These modifications correlate with alterations in antigen processing efficiency, MHC class II peptide binding, and DM editing activity [30].

Finally, due to insulin resistance and hyperglycemia, β-cells experience increased secretory stress. This leads to a gradual decline in pancreatic function in patients with diabetes, which further exacerbates the progression of diabetes [31]. The accumulation of harmful lipids, particularly sphingolipids such as ceramides, in β-cells and other tissues, including liver tissues, is closely associated with this deterioration [32]. Recent clinical trials, building on animal studies, have confirmed that ceramides primarily affect the pathogenesis of T2DM through their impact on β-cell functionality [33].

#### Cellular stress responses and metabolic dysfunction

Cellular stress responses not only serve as a biological phenomenon associated with cellular reactions to adverse environmental stimuli such as malnutrition, hypoxia, or toxin exposure but also play a pivotal role in the complex etiology of T2DM and its complications [34]. These stress responses have implications for pancreatic β-cell functionality and may exacerbate the pathophysiological mechanisms underlying T2DM and its associated complications. In the context of T2DM, hypoxia-inducible factor (HIF)-1α plays a central role in regulating pancreatic β-cells, particularly under sustained metabolic stress35. Elevated glucose levels and insulin resistance compromise HIF-1α stability, further diminishing HIF-1α expression, a mechanism that is exacerbated by elevated 2-methylglyoxal levels [36]. Additionally, the lipid milieu, particularly the metabolism of fatty acids such as palmitate, exerts dual effects on HIF-1α stability and endothelial function. Experimental data indicate that HIF-1α inhibitors such as PX-478 can significantly ameliorate β-cell dysfunction under hyperglycemic conditions [35, 36].

Endoplasmic reticulum (ER) stress, commonly observed in metabolic diseases, is characterized by excessive protein unfolding or misfolding and is associated with conditions such as diabetes, insulin resistance, and obesity [37]. In T2DM, this stress precipitates a marked reduction in viable β-cell mass, a critical determinant of insulin secretion [38]. Consequently, ER stress-induced damage to β-cells precipitates their functional deterioration, thereby intensifying T2DM progression [38]. Furthermore, the unfolded protein response (UPR) within the ER is pivotal for protein homeostasis; however, its perturbation is intimately associated with diminished pancreatic β-cell survival [39, 40]. As a key sensor of ER stress, protein kinase RNA-like endoplasmic reticulum kinase (PERK) orchestrates stress responses at the interface between the ER and mitochondria, forming a PERK-mitochondrial axis that maintains the functional integrity of both organelles [41]. Under conditions of high glucose or exposure to saturated fatty acids, the PERK pathway is predominantly activated, thereby promoting excessive mitochondrial fusion and preventing premature fission and apoptosis [41]. Through the activation of beneficial pathways, such as the PERK-OGT, PERK-TFEB, and PERK-NRF2 pathways, PERK maintains mitochondrial dynamics, metabolism, and quality control, providing protection in metabolic disease models [41]. Recent studies have revealed a dramatic increase in reactive oxygen species (ROS) in cardiomyocytes under high-glucose conditions [42]. The surge in ROS is attributed to the NADPH oxidase NOX2, whose activity is regulated by O-GlcNAcylation at the CaMKIIδ S280 site, suggesting a novel mechanism that could exacerbate macrovascular complications in diabetes [42]. In the pathogenesis of T2DM, reductive stress, a specialized form of cellular stress, impacts cellular signaling by reducing physiological levels of ROS [43]. Under reductive stress conditions, cells mitigate the stress status by ubiquitinating the mitochondrial gatekeeper FNIP1 [43].

Cellular energy metabolism is at the core of biological activity and principally relies on glucose, fatty acids, and amino acids for energy supply [44]. Aberrant cellular energy metabolism not only induces vascular injury but also activates a multitude of cellular stress pathways, affecting cell survival and function. Specifically, this process includes the activation of protein kinase C pathways, increased activity of endothelial guanylate cyclase, and uncoupling of endothelial nitric oxide synthase (eNOS), thereby elevating ROS levels [36]. Concurrently, lipid metabolism, particularly that of palmitate esters, exerts dual effects on HIF-1α and endothelial function, thereby exacerbating macrovascular complications in T2DM patients [36]. Utilizing the HIF-1α inhibitor PX-478 under high-glucose conditions can significantly improve β-cell function, including baseline insulin release and intracellular Ca2+ oscillation adjustments [35]. Glucose metabolism is central to T2DM, and its intracellular overload leads to hyperactivation of various biochemical pathways. This process enhances ROS generation and depletes NADPH, further intensifying oxidative stress. Amino acid metabolism also plays a key role in T2DM and its macrovascular complications. Specifically, branched-chain amino acids, such as glutamine, are emerging as new biomarkers for risk prediction. Glutamine is negatively correlated with body mass index (BMI) and the homeostatic model for insulin resistance, and glutamine metabolism influences macrophage polarization and atherosclerotic plaque formation [45].

### Alterations in the vascular microenvironment induced by diabetes

#### Endothelial dysfunction

Healthy ECs form the innermost layer of blood and lymphatic vessels, which serve as multifunctional organs with systemic and tissue-specific functions that regulate the supply of oxygen and nutrients, the migration of immune cells, and the inflammatory process [46]. Endothelial dysfunction in diabetes is characterized by impaired endothelium-dependent vasodilation, elevated oxidative stress, chronic inflammation, increased leukocyte adhesion, increased permeability, and cellular senescence [8]. Clinically, endothelial dysfunction manifests early during diabetes. It is closely associated with poor prognosis and leads to insulin resistance, microvascular complications, and atherosclerosis. Notably, impaired endothelium-dependent vasodilation can be measured even before any morphological changes in the vascular wall are evident, suggesting that reduced nitric oxide (NO) production and/or activity may be a critical early event in vascular pathogenesis [47]. Specifically, elevated levels of beta-amyloid protein 42 and endothelin-1 have been found to influence the activity of eNOS, cyclic guanosine monophosphate, and protein kinase G, thereby exacerbating endothelial dysfunction and chronic inflammation [47].

Impaired angiogenesis in diabetes patients is a key process contributing to macrovascular complications. Epigenetic mechanisms, particularly the long noncoding RNA LEENE (endothelial NO synthase enhancer), play a central role in this process [48]. Reduced expression of LEENE under diabetic conditions leads to a decrease in endothelial angiogenic capabilities and compromised perfusion in ischemic limbs [48]. Moreover, endothelial microparticles serve as early surrogate markers for vascular dysfunction; their release is elevated under diabetic conditions, especially due to increased cellular apoptosis [49]. Perivascular adipose tissue serves as a crucial modulator of vascular homeostasis, releasing various adipokines, chemokines, and growth factors [50]. Obesity and T2DM result in perivascular adipose tissue dysfunction, amplifying the inflammatory response in the vascular wall and leading to impaired function in both endothelial and smooth muscle cells [50]. Asprosin, a recently discovered adipokine, has been demonstrated to have significant regulatory effects on metabolism [51]. Mechanistically, it induces endothelial-to-mesenchymal transition through the activation of the TGF-β signaling pathway, leading to further vascular damage [51]. Specifically, studies have shown that asprosin directly induces endothelial-to-mesenchymal transition in human umbilical vein ECs and that this effect is TGF-β dependent.

#### Hemorheological variations and coagulation abnormalities

Hypercoagulable states and excessive platelet activation serve as crucial factors contributing to vascular complications in patients with diabetes, resulting from an interplay of multiple mechanisms [52, 53]. Under hyperglycemic conditions, platelet activation is notably enhanced and is associated with increased activity of the IRE1α-XBP1 pathway in the UPR. In addition, various UPR pathways, such as the PERK and ATF6 pathways, are selectively induced under diverse stress conditions, indicating their distinct roles in platelet function [54]. For instance, the absence of PERK is correlated with increased platelet aggregation and apoptosis, highlighting its potential impact on thrombotic risk in T2DM patients [54]. This further accelerates the progression of arterial atherosclerosis, which may ultimately result in severe cardiovascular events such as myocardial infarction or stroke [52, 53]. Thromboxane A2 and branched-chain amino acids (BCAAs) are considered key biochemical mediators that facilitate these pathological alterations [55]. Thromboxane A2 is a potent pro-thrombotic agent produced by platelets via the COX-1 pathway [55]. It is capable of inducing platelet activation and vascular constriction, thereby exacerbating endothelial dysfunction [55]. Recent studies have emphasized the role of BCAAs in platelet activation and thrombus formation; specifically, the breakdown of BCAAs significantly increases platelet activity and arterial thrombosis in mice [56]. The valine metabolite α-ketoisovalerate has been identified as a primary factor underlying these effects [56]. Platelet-derived extracellular vesicles (EVs) are implicated in the worsening of atherosclerotic complications [57]. These EVs, which are released from monocyte‒platelet aggregates under the influence of tumor necrosis factor-α (TNF-α), possess proinflammatory properties that exacerbate atherosclerosis [57]. Importantly, antiplatelet drugs, such as aspirin and P2Y12 inhibitors, can modify the proinflammatory characteristics of these EVs [57].

Hyperreactive platelets are often observed in patients with diabetes, suggesting a potential link between glycemic homeostasis and platelet reactivity [58]. Research has shown that factors from β-cells can stimulate platelet activity and that platelets selectively localize to the endothelium of pancreatic islets [58]. Elimination of platelets and primary platelet adhesion or activation pathways consistently result in impaired glucose tolerance and reduced circulating insulin levels [58]. Furthermore, platelet-derived lipids have been found to promote insulin secretion, and 20-HETE has been identified as the principal factor that enhances β-cell functionality [58]. Additionally, secreted modular calcium-binding protein 1, regulated by microRNA-223, has been identified as a novel coagulation activation protein in platelets [59]. Elevated levels of SMOC1 have been observed in the platelets of T2DM patients and are directly related to high thrombin reactivity [59]. Recent studies have also shown that neutrophils interact with megakaryocytes in the bone marrow to expedite platelet generation through a mechanism termed “nibbling”, increasing the risk of recurrent ischemia post-myocardial infarction [60].

Continuous remodeling of hematopoietic cell lineages contributes to the thrombotic risk associated with T2DM. These mechanisms may have broader implications in the context of chronic diseases [61]. These studies elucidate the multifaceted roles of platelets in T2DM patients, extending beyond the traditional domains of thrombogenesis and vascular complications. These findings highlight the potential for targeting specific platelet pathways not only to mitigate thrombotic risks but also to improve metabolic control in T2DM patients.

Although abundant research exists on antithrombotic treatment in vascular disease patients, diabetes-specific randomized controlled trials, particularly those focusing on secondary prevention, are still lacking [62]. Vascular risk in patients with diabetes varies by individual and depends on the duration of diabetes and its complications, adding to the complexity and hindering the formulation of clear guidelines.

### Comprehensive impacts and intersecting pathways

#### Glycolysis and multipathway impacts

Glycolysis and gluconeogenesis, along with tricarboxylic acid cycle metabolites, are intricately linked to T2DM [63]. Glucokinase serves as the initial enzyme in the glycolytic pathway, catalyzing the conversion of glucose to glucose-6-phosphate [64]. Although glucokinase is considered a potential therapeutic target for ameliorating hyperglycemia in patients with diabetes, drug development targeting this enzyme is associated with numerous challenges [64]. One substantial issue is that activators of glucokinase may excessively stimulate glycolysis in pancreatic β-cells, leading to β-cell failure [64]. Furthermore, metabolic byproducts of glycolysis can induce mitochondrial dysfunction and mTORC1 activation in diabetic β-cells [31]. This further affects insulin synthesis and secretion, thereby perpetuating a hyperglycemic state [31]. Hyperactive glycolysis is a distinct metabolic feature of aging and diabetic β-cells and is closely related to β-cell hyper-responsiveness to glucose and impaired cellular identity [65]. Specifically, enhanced glycolytic activity has been observed through the increased expression of an enzyme called nicotinamide mononucleotide adenylyl transferase 2, which further affects β-cell insulin secretion and molecular identity [65]. Interestingly, β-cell function and identity can be restored by inhibiting the activity of this enzyme or slowing glycolysis [65]. Insulin resistance often precedes diabetes and frequently coexists with it. Specifically, insulin coordinates glucose absorption in adipocytes through the mTORC2/AKT signaling pathway and GLUT4 translocation to the cell membrane. Upon entering adipocytes, glucose undergoes glycolysis [66]. This pathway serves as a central hub for multiple metabolic pathways, either supporting or impairing adipocyte function, blood glucose levels, and overall metabolic balance.

Glycolysis is a fundamental metabolic pathway that is also closely associated with inflammation, oxidative stress, and endothelial function [67]. Mechanistic studies have shown that aerobic glycolysis increases proinflammatory gene expression and that hyperglycemia promotes proinflammatory gene expression [13]. This metabolic shift leads to elevated levels of metabolic intermediates, such as succinate and malate. These metabolites act as antagonists of histone and DNA demethylases, further exacerbating inflammatory responses. Activation of succinate receptor 1 may promote the migration and activation of inflammatory cells, thereby exacerbating chronic inflammation [68]. Concurrently, activation of this receptor in ECs may affect vascular contractile function, further aggravating endothelial dysfunction and increasing the susceptibility of patients with diabetes to macrovascular complications, such as arteriosclerosis and cardiovascular diseases (CVDs) [68]. Next, we focus on IgG *N*-glycosylation, a posttranslational modification that may alter the risk of T2DM and CVD through its immunomodulatory functions [69]. Further research has indicated that AGEs and their interaction with RAGE drive proinflammatory/oxidative pathways, causing molecular, cellular, and vascular damage [27]. Moreover, RAGE can bind to multiple proinflammatory ligands that accumulate in diabetic tissues, exacerbating vascular damage. Specifically, the cytoplasmic tail of RAGE plays a crucial role in RAGE signaling by interacting with Diaphanous-1. Researchers have successfully inhibited this interaction using the small-molecule antagonist RAGE229, which is capable of alleviating diabetic complications [70]. RAGE229 not only reduces the concentrations of the inflammatory cytokines TNF-α, interleukin-6 (IL-6), and CCL2/JE-MCP1 but also alleviates pathological and functional indicators of diabetic complications.

Methylglyoxal (MGO) is a highly reactive dicarbonyl compound primarily derived from glycolysis that is highly reactive [28, 67]. With increased glycolysis, MGO levels also increase. MGO mainly forms as a byproduct of glycolysis, and its accumulation leads to nonenzymatic glycation of proteins and DNA, subsequently leading to the formation of AGEs [71, 72]. As persistent modifications, AGEs continue to induce late diabetic complications even after blood glucose normalization, an effect termed "metabolic" (or better, the legacy effect) [67]. This residual risk is attributed to two key factors: the presence of additional risk factors independent of glycemic control and the prolonged imbalances caused by uncontrolled glycemia, known as metabolic memory or the legacy effect [73]. MGO and AGEs negatively impact the structure and function of vessels and organs, contributing to organ damage [71, 72]. In patients with diabetes, the detoxification system for MGOs, specifically the glyoxalase system, is impaired, leading to increased MGO concentrations and glycotoxic burden [67]. The androgen/androgen receptor axis plays a role in regulating glycolysis and the progression of diabetes. Particularly in male model animals, disruption of this axis enhances glycolytic activity by up to 30%, accelerating the development of diabetes [74]. Recent research has shown that decreases in FOXO1 levels enhance glycolysis-dependent DNA repair through PFKFB3, thereby mitigating endothelial oxidative stress damage induced by hyperglycemia [75]. This discovery highlights the importance of glycolysis in DNA repair and vascular function under hyperglycemic conditions. In this process, PFKFB3 not only promotes glycolysis but also directly participates in DNA repair [75]. Under genotoxic stress, PFKFB3 relocates to oxidative stress-induced DNA damage sites and promotes DNA repair through interactions with the MRE11-RAD50-NBS1 complex-ATM pathway [75]. This mechanism provides a new therapeutic target for diabetic vascular complications and further emphasizes the complex role of glycolysis in macrovascular complications in diabetes.

#### Oxidative stress and inflammatory responses

In the context of macrovascular complications in diabetes mellitus patients, oxidative stress serves as a pivotal factor that significantly influences both the onset and progression of the disease. Oxidative stress often induces inflammatory responses, which in turn generate additional ROS, establishing a vicious cycle. According to recent research, patients with diabetes frequently experience hyperglycemia, which leads to endothelial dysfunction and a marked increase in oxidative stress [76]. Under hyperglycemic conditions, the expression levels of the Nox1 and Nox4 genes as well as the production of ROS are elevated in human vascular smooth muscle cells (VSMCs), subsequently resulting in the overexpression of inflammatory markers, such as MCP-1 and VCAM-1, and fibrotic markers, such as CTGF [76]. This oxidative stress-induced inflammatory response is mediated through the activation of the NADPH oxidase/NF-κB signaling pathway.

Macronutrients play an indisputable role in elevating the risk of T2DM [77]. Protein carbonylation is an irreversible protein modification triggered by oxidative stress. In diabetes patients, increased oxidative stress may increase protein carbonylation, leading to cellular damage. This damage could contribute to the development of macrovascular complications, including arteriosclerosis [28]. Concurrently, the micronutrient iron is a risk determinant not only for pathological excess or deficiency but also within its broad physiological range [77]. Excessive free iron can induce oxidative stress, further affecting the state of macrophages [77, 78]. This potentially exacerbates insulin resistance and causes macrovascular damage [77, 78].

Inflammation is considered a significant risk marker for systemic atherosclerosis and is also a prominent feature of vascular complications induced by diabetes [2, 79]. Adipocytokines, such as TNF-α and IL-6, negatively impact insulin signaling by activating inflammatory pathways and ER stress [80]. This state of insulin resistance not only disrupts glucose homeostasis but also further impairs EC function, leading to dysregulation of vascular constriction and dilation [80]. This dysregulation elevates the risk of cardiovascular and cerebrovascular diseases, thereby exacerbating the overall pathological condition of T2DM patients. Concurrently, studies have shown that in patients with T2DM, IL-6 is significantly associated with cardiovascular and renal outcomes, further exacerbating chronic inflammation and endothelial dysfunction [81]. Specifically, for each doubling of baseline IL-6 levels, the risk for cardiovascular and renal outcomes increases by 14% and 21%, respectively [81]. During T2DM progression, bone marrow ECs and hematopoietic stem and progenitor cells critically regulate chronic inflammation and endothelial dysfunction [82]. Reduced Cxcl12 expression in these cells under diabetic conditions triggers hematopoietic stem and progenitor cell proliferation and elevates proinflammatory myeloid counts. This imbalance leads to the excessive release of proinflammatory cells and aberrant progenitor cell differentiation, ultimately resulting in β-cell dysfunction in T2DM patients [82].

#### Impact of epigenetic factors

Epigenetic modifications play pivotal roles in the pathophysiological mechanisms underlying macrovascular complications in T2DM patients [83]. Specific epigenetic modifications, particularly alterations in DNA methylation, have been identified in tissues, such as pancreatic islet tissue and adipose tissue, of T2DM patients [83]. These modifications are influenced by both genetic and nongenetic factors, including obesity and lifestyle factors [83]. Environmental factors associated with T2DM, such as lifestyle and dietary habits, also impact these epigenetic states, including DNA methylation and histone modifications that modulate gene transcription in response to environmental stimuli [84].

Recent studies further highlight that the downregulation of DJunD gene expression in T2DM patients is linked to oxidative stress, cardiac dysfunction, and inflammatory responses [85]. This downregulation involves intricate epigenetic mechanisms, including DNA methylation and miRNA-mediated posttranscriptional repression [85]. Macrophage phenotype dysregulation serves as a key driver in various diseases, including atherosclerosis and T2DM [12]. Exposure to inflammatory cytokines and lipids induces epigenetic heterogeneity in macrophages, leading to plaque formation through complex transcriptional changes influenced by their microenvironment [12]. Moreover, HIFs in T2DM patients also modulate the expression and/or activity of epigenetic regulatory factors in response to hypoxia, underscoring the multifaceted role of epigenetics in T2DM and its macrovascular complications [86, 87].

Epigenetics is also linked to the phenomenon of metabolic memory observed in clinical trials and animal studies [84]. Recent research has confirmed that in metabolic memory, the glycemic control achieved in the early stages of diabetes significantly determines the trajectory of future vascular health [88]. Specifically, HbA1c levels exceeding 6.5% or 7% are significantly associated with an increased risk of developing late-stage MACE, with increased risk ranging from 19 to 64% [88]. Additionally, studies of epigenetic dosage have identified two functionally distinct β-cell subtypes, namely, βHI and βLO, which are differentiated by varying levels of the histone marker H3K27me3 [89]. This research emphasizes the clinical importance of this epigenetic marker as a potential target for secondary prevention strategies for T2DM [89].

## Protective mechanisms against macrovascular complications in type 2 diabetes mellitus patients

### Systemic protective factors

In patients with diabetes mellitus, a diverse array of systemic and tissue-specific protective factors counteract the harmful impact of toxic metabolites, attenuating the risk of long-term complications [90]. Within the context of secondary prevention of macrovascular complications in T2DM patients, these protective agents, which include antioxidative enzymes and anti-inflammatory cytokines, pervasively manifest in both the circulatory system and various tissues and are complemented by enhanced glycemic regulation, metabolic memory phenomena, and a spectrum of circulating bioactive molecules [90].

Primarily, glycemic control serves as a foundational preventive strategy. Metabolic memory effects and fasting glucose levels exert direct impacts on multiple systems. For instance, the quantity and functionality of endothelial progenitor cells (EPCs), which play pivotal roles in vascular repair and angiogenesis in peripheral vascular disease patients, are inversely correlated with fasting glucose levels [91]. These findings suggest that metabolic control in diabetes may directly influence EPC count and function [91]. Sirtuins are NAD-dependent protein deacetylases known for their protective role in diabetes and are also noteworthy [92]. Specifically, SIRT2 has been identified as a crucial regulator of glucose metabolism and β-cell function [92]. It modulates key metabolic proteins, such as GKRP and ALDOA, by deacetylation, thereby influencing glycolytic pathways and insulin secretion.

The transcription factor RUNX1 is associated with chronic inflammation and immune responses and is a potential therapeutic target for high glucose-induced trained immunity [13]. RUNX1 collaborates with other key factors, such as PU.1, to regulate macrophage colony-stimulating factor receptors, thereby affecting macrophage survival, differentiation, and proliferation. Pharmacological inhibition of RUNX1 provides a potential therapeutic strategy for high glucose-induced trained immunity [13]. Similarly, in β-cells, inflammatory signals lead to mitochondrial damage, triggering bioenergetic impairment and apoptotic mechanisms. Identifying protective responses against inflammation could offer clues for new therapeutic targets. Researchers have reported that mitochondrial autophagy is a protective response against inflammatory stress in both human and rodent β-cells [93].

Recently, N-terminal pro-brain natriuretic peptide (NT-proBNP) has garnered increasing attention as a novel systemic protective factor. Elevated levels of NT-proBNP, traditionally linked to heart failure, inversely correlate with the risk of developing T2DM due to the role of NT-proBNP in metabolic regulation [94, 95]. Moreover, this biomarker not only orchestrates cardiovascular homeostasis but also modulates glucose and lipid metabolism, enhancing insulin sensitivity and reducing inflammation through its effects on adipocytes and adiponectin secretion [94, 96]. Consequently, higher NT-proBNP levels confer protection against T2DM onset. Interestingly, this inverse relationship extends to the domain of diabetes-related complications [94, 95]. While elevated NT-proBNP levels indicate a decreased risk of T2DM, they are associated with an increased risk of both microvascular and macrovascular complications in individuals with diabetes [94, 95]. This dual role of NT-proBNP suggests that NT-proBNP levels reflect broader physiological processes that influence the risk of diabetes and its complications, underscoring the complexity of metabolic and cardiovascular interplay in diabetes management. The distinct patterns observed in men and women regarding NT-proBNP levels and diabetes risk further emphasize the need for sex-specific considerations in diabetes risk assessment and management strategies.

In summary, these systemic protective factors, including RUNX1, EPCs, and NT-proBNP, not only enrich our understanding of the protective mechanisms in T2DM and its macrovascular complications but also hold promise as prospective biomarkers for risk stratification and tailored therapeutic interventions.

### Tissue-specific protective factors

In addition to systemic protective elements, emerging evidence underscores that individual tissues may harbor unique protective responses, thereby contributing to the diverse pathological manifestations observed across various complications [90]. At the tissue level, localized responses generate protective factors that mitigate vascular complications through antioxidative and anti-inflammatory mechanisms [90]. These processes primarily involve the maintenance and repair of vascular endothelial and smooth muscle cell functions as well as the modulation of energy metabolism pathways to slow the progression of vascular complications [90].

EC dysfunction is a pivotal factor in macrovascular complications. Elevated expression of Quaking-7 under hyperglycemic conditions correlates with compromised cellular barriers and angiogenic capacity [97]. Mechanistic studies have revealed that LEENE interacts with key components of the RNA polymerase II-associated factor complex, LEO1, and the critical transcription factor MYC, thereby promoting the transcription of proangiogenic genes such as KDR and NOS348. Recent research has elucidated the crucial role of ECs and NONO in improving systemic bioenergetics and reducing atherosclerotic risk [98]. For instance, enhanced insulin responsiveness and NO production in ECs facilitate the differentiation of peripheral vascular progenitor cells (PPCs) into brown adipose tissue (BAT), thereby improving systemic energy expenditure and weight loss [98]. Moreover, the lipid 12,13-diHOME significantly elevates the NO-induced increase in BAT, improving endothelial dysfunction and reducing atherosclerotic risk [98]. Under hyperglycemic and hyperlipidemic conditions, basic fibroblast growth factor alleviates diabetes-associated endothelial dysfunction and angiogenic defects by maintaining S-nitrosylation balance and suppressing inflammation [99]. This mechanism is further facilitated by increased activity of NOeNOS and thioredoxin [99]. Under conditions of nutrient excess, adipocytes balance lipid and glucose influx through growth and proliferation as a primary tissue-specific protective response [100]. These responses activate resident immune cells to support tissue function and restore homeostasis. However, these protective mechanisms become insufficient under the long-term impact of chronic nutrient excess [100].

In terms of tissue-protective factors, the roles of FOXO1 and PFKFB3 in ECs are particularly noteworthy. These molecular pathways not only promote glycolysis but also directly participate in DNA repair, thereby alleviating diabetes-associated vascular damage [75]. Additionally, mitsugumin 53 (MG53), an essential component of the cellular membrane repair mechanism, exhibits significant cardioprotective effects. However, MG53 also impacts insulin signaling through its E3 ligase activity and myokine function. Recent research has identified a specific site mutation (S255A) that eliminates the adverse effects of MG53 on insulin signaling while preserving its cardioprotective function [101]. Acidic fibroblast growth factor (aFGF) has garnered attention for its potent antioxidative effects. aFGF significantly reduces the generation of mitochondrial superoxide anions and achieves this protective effect through the Wnt/β-catenin/c-Myc axis [102].

### Insulin and its dual role in vascular tissue

In the treatment and management of T2DM, insulin plays complex dual roles, encompassing both protective effects on vascular tissues and potential adverse effects [90]. Under normal conditions, insulin facilitates vasodilation and enhances vascular function by activating eNOS and provides protection by reducing inflammation levels [90]. These actions contribute to maintaining cardiovascular health and preventing diabetes-related micro- and macrovascular complications.

However, with the development of insulin resistance, the emergence of compensatory hyperinsulinemia has exacerbated pathological processes, such as the proliferation of vascular smooth muscle cells and atherosclerosis, in turn increasing the risk of cardiovascular events [90]. This shift occurs at a critical point when the protective effects of insulin are overtaken by its potential harmful effects. This critical point is determined by several factors, including an individual's insulin sensitivity, insulin concentration, and state of chronic inflammation [90].

## Preventive strategies for macrovascular complications in type 2 diabetes mellitus patients

The primary clinical objective for patients with T2DM is to prevent or delay the progression of complications and improve quality of life [103]. While glycemic control has long been considered the cornerstone for mitigating the risk of macrovascular complications in T2DM patients, recent studies have revealed that cardiovascular complications remain significantly prevalent even under stringent glycemic control [13]. In addition to traditional glycemic management, alternative strategies must be explored to prevent diabetes-related cardiovascular complications. A comprehensive approach encompassing biomarker monitoring and management, lifestyle interventions, and pharmacological prevention has proven effective (Table 1).

### Biomarker monitoring and management

Current guidelines for T2DM management are gradually shifting toward personalized glycemic targets and precision medicine paradigms [104]. This shift focuses not only on achieving significant glycemic control but also on optimizing safety and nonglycemic benefits to enhance the added value of preventing macrovascular complications [104]. Within this framework, biomarkers have emerged as critical factors warranting clinical attention [104].

In the management of T2DM, traditional biomarkers such as HbA1c, blood pressure, lipid levels, heart rate, body weight, and serum uric acid, along with their variabilities, are of paramount importance [105]. In particular, HbA1c is the most commonly used parameter for assessing glycemic control [106]. However, modern approaches in glycemic management extend beyond achieving optimal HbA1c levels as early as possible. These strategies are aimed at reducing postprandial hyperglycemia and glycemic variability as well as maximizing the duration of time in the near-normal glycemic range [107]. Importantly, HbA1c variability is a predictor of cardiovascular complications in patients with T2DM, regardless of whether glucose levels have reached the target. Furthermore, variabilities in other risk factors, such as blood pressure, lipid profile, heart rate, body weight, and serum uric acid levels, also play a significant role in the development of diabetes-related complications [105]. The variability of each risk factor and their combined effects may amplify the risk of atherosclerotic cardiovascular disease (ASCVD) in T2DM patients.

Notably, a range of biomarkers, including relative leukocyte telomere length, serum endotrophin, and circulating palmitoyl sphingomyelin, have been identified as risk factors for cardiovascular complications in T2DM patients [108–110]. These biomarkers offer robust tools for more precisely assessing cardiovascular risk and providing personalized management recommendations. In addition, protein carbonyls, hydroxymethylfurfural and fibrinogen stand out for their validated use in assessing an individual's resistance to macrovascular complications, with their altered levels in T2DM patients mirroring the heightened chronic inflammation and oxidative stress that are central to the pathogenesis of such complications [28]. Recent studies have revealed significant correlations between novel inflammatory biomarkers, such as the neutrophil-to-high-density lipoprotein ratio, monocyte-to-high-density lipoprotein ratio, platelet-to-high-density lipoprotein ratio, systemic immune-inflammation index, systemic inflammatory response index, aggregate inflammatory index, and peripheral arterial disease, and T2DM [111]. Notably, the combined model of the neutrophil-to-high-density lipoprotein ratio and systemic inflammatory response index has demonstrated the highest predictive value for T2DM-PAD [111]. These markers identified in T2DM-PAD patients independently correlate with disease severity and can be readily assessed through standard laboratory indices, offering significant potential for clinical application.

In the context of diabetes-related vascular complications, emerging evidence suggests that in Australia's indigenous population, dysregulated HDL-miRNA profiles could undermine HDL functionality, potentially serving as biomarkers for the exacerbation of these complications [112]. Asprosin, a recently discovered adipokine, has been identified as an independent risk factor for PAD in T2DM patients. Elevated circulating levels of asprosin have been found to be significantly correlated with PAD and negatively correlated with the diagnostic marker the ankle-brachial index [51]. Cysteine-rich angiogenic inducer 61 (Cyr61) is significantly correlated with PAD in T2DM patients [113]. Cyr61, a 40-kD secretory protein, has been shown to play a crucial role in regulating cellular physiological processes [113]. This study indicated that Cyr61 levels are significantly increased in PAD patients with T2DM and are positively correlated with disease severity [113]. Additionally, urinary thromboxane A2 metabolites are considered noninvasive biomarkers for elevated cardiovascular risk and are valuable tools for secondary prevention of T2DM [55].

In summary, the integration of personalized medicine and precision mechanisms offers a promising avenue for effectively preventing cardiovascular and macrovascular complications in patients with diabetes. This requires a multidisciplinary approach, including basic research, clinical trials, and the development of personalized treatment plans [114]. Future research is expected to reveal genetic markers that can predict the likelihood of enhanced responses to antidiabetic medications and the onset of complications [104].

### Lifestyle interventions

Lifestyle interventions constitute a critical component of the management of T2DM and its macrovascular complications. In addition to genetic factors, unhealthy dietary habits, physical inactivity, and weight gain disrupt circadian physiological processes, leading to metabolic dysregulation and elevating the risk of T2DM and its complications [115]. Comprehensive lifestyle management, encompassing medical nutrition therapy, weight reduction, and physical activity, serves as an effective strategy for mitigating the risk of macrovascular complications [5].

#### Medical nutrition therapy

The primary goal of medical nutrition therapy used in the management of T2DM is to achieve optimal metabolic control—maintaining blood glucose and lipid levels within recommended ranges—to reduce the risk of macrovascular complications [116]. In medical nutrition therapy, a balanced diet and judicious micronutrient supplementation are considered effective strategies [117]. Various diets, including the Mediterranean and Paleolithic diets and low-carbohydrate, high-protein, vegetarian, and nut-rich diets, are recommended [5, 63, 116, 118]. Conversely, a poor diet involving the consumption of, for example, sugary beverages, foods high in sugar and fat, and minimal fresh fruits and vegetables increases the risk of weight gain and T2DM complications [119–121]. Disruptions in nutrient sensing and responses to internal levels of nutrients, such as glucose, lipids, and amino acids, are closely associated with metabolic diseases, such as obesity, T2DM, and other metabolic syndromes and their complications [122]. Recent meta-analyses of 884 randomized controlled trials provide moderate-to-high-quality evidence that micronutrients, such as n-3 fatty acids, folic acid, and coenzyme Q10, can reduce the risk of macrovascular diseases in T2DM patients [123]. However, supplementation with certain micronutrients, such as β-carotene, may increase all-cause mortality and CVD risk [123]. BCAAs play pivotal roles in protein synthesis, energy metabolism, and cellular regulation. However, chronic accumulation of BCAAs, induced by either dietary or genetic factors, is associated with metabolic dysregulation, insulin resistance, and increased cardiovascular risk, particularly in patients with obesity and diabetes [117, 124]. When micronutrient supplementation is recommended for T2DM patients for cardiovascular complication prevention, various factors, including the type, dosage, and timing of nutrient intake and overall health status, should be carefully considered.

Recent studies have revealed the impact of a high-fat diet on the adaptive process of β-cell function, the transition from compensation to decompensation, and how dietary interventions can reverse this process [125]. Dietary interventions in the prediabetic stage can fully restore β-cell function and significantly reverse the chromatin and transcriptomic reprogramming induced by a high-fat diet [125]. Healthy diets can also reverse hepatic insulin resistance and steatosis caused by ER-mitochondrial miscommunication, suggesting a potential therapeutic target for restoring metabolic balance [126]. Furthermore, research indicates that a caloric restriction of approximately 14% in healthy populations can significantly improve thymic output and induce transcriptomic reprogramming in adipose tissue within two years via pathways that regulate mitochondrial bioenergetics, anti-inflammatory responses, and longevity [127]. Specifically, caloric restriction can inhibit the expression of the platelet-activating factor acetylhydrolase gene, which is associated with slowing thymic atrophy, reducing inflammation, and improving metabolic health [127].

#### Weight reduction and increased physical activity

The clinical demarcations for overweight and obesity are a BMI exceeding 25 kg/m^2^ and 30 kg/m^2^, respectively, and excess adiposity is intricately linked with adverse cardiovascular outcomes [5]. The pathophysiological cascade initiated by excess adiposity extends beyond mere weight gain, engendering hypertension, dyslipidemia, endothelial dysfunction, and a heightened inflammatory milieu [5, 128]. Central to these processes is metabolic turmoil within adipose tissue, characterized by hypoxia, perturbed protein folding, and an increase in circulating free fatty acids, which collectively precipitate systemic inflammatory pathways [5, 40, 128]. This insidious inflammatory state not only fosters insulin resistance and pancreatic β-cell impairment but also accelerates the progression of T2DM and its macrovascular sequelae [5, 128]. Recently, both the American Diabetes Association and the European Association for the Study of Diabetes convened a panel to update their consensus statement on adult T2DM management, emphasizing the importance of weight management within the overall approach to diabetes care [129]. Similarly, the American Heart Association underscores that moderate and sustained weight loss can offer substantial cardiovascular benefits [5]. This perspective is supported by recent Mendelian randomization studies, highlighting the significance of effective weight management in reducing macrovascular complication risks in T2DM patients [130]. Different types of adipose tissue contribute variably to vascular dysfunction and subsequent CVD. The accumulation of visceral adipose tissue is associated with immune cell infiltration and increased secretion of vasoconstrictive mediators, thereby elevating the risk of vascular complications [131, 132]. Weight reduction benefits T2DM patients in terms of glycemic control, concentric left ventricular remodeling, aortic stiffness, and reduced risk of heart failure [114, 133].

Increased physical activity and exercise have been shown to reduce weight and improve various parameters, including blood glucose, lipids, blood pressure, insulin sensitivity, and inflammatory biomarkers, in a dose-dependent manner [5, 118, 134]. Clinical studies indicates that various types of exercise interventions, including dynamic aerobic exercise, combined aerobic and resistance training, dynamic resistance exercise, and mind–body therapies, can offer significant health benefits to diabetic patients [135, 136]. The median duration of exercise interventions at 135 min per week demonstrates that increasing the level of physical activity can effectively reduce the risks of all-cause mortality, cardiovascular mortality, myocardial infarction, and stroke. Particularly, a high level of physical activity, compared to a low level, can significantly reduce the incidence rate of total cardiovascular diseases by 16%, coronary heart disease by 16%, cerebrovascular events by 26%, and the occurrence of heart failure by 24% [135, 136].

While the positive impacts of exercise are widely acknowledged, the underlying molecular mechanisms are not fully understood. In both acute and/or chronic exercise states, signaling molecules are released through endocrine, paracrine, or autocrine pathways. Various organs, cells, and tissues, such as skeletal muscles (myokines), heart (cardiokines), liver (hepatokines), white adipose tissue (adipokines), brown adipose tissue (baptokines), and neurons (neurokines), are involved in the release of these signaling molecules [134]. Recent mechanistic studies have identified specific signaling molecules, such as *N*-lactoylphenylalanine (Lac-Phe), that are induced by exercise and act as appetite and obesity suppressors [137]. Long-term administration of Lac-Phe has demonstrated potential for maintaining energy balance by regulating food intake and weight control [137]. Other studies have revealed that exercise improves the metabolic rate and resistance to obesity by activating mild mitochondrial stress responses in hypothalamic POMC neurons [138]. A recent study provided the first empirical evidence that early moderate or high-intensity exercise can effectively prevent and ameliorate diabetic heart disease, partially through the regulation of cardiovascular-specific microRNAs [139].

In addition to lifestyle interventions, drug intervention and surgical approaches have shown superiority in weight reduction and prevention of macrovascular complications in T2DM patients. In managing T2DM, semaglutide, a GLP-1 receptor agonist, has demonstrated significant efficacy in weight management alongside glycemic control [140, 141]. The STEP 2 and PIONEER PLUS trials elucidated the benefits of semaglutide at dosages up to 2.4 mg subcutaneously weekly and 25 mg to 50 mg orally daily, revealing marked reductions in body weight and HbA1c levels [140, 141]. These studies highlight the dual effects of semaglutide, which results in both hyperglycemia and obesity in T2DM patients and tolerable gastrointestinal side effects. This evidence positions semaglutide as a key therapeutic option, expanding the role of GLP-1 receptor agonists beyond glucose regulation to encompass weight management, thereby enriching the treatment arsenal for T2DM with a focus on comprehensive metabolic health improvement.

Beyond pharmacological treatments, bariatric surgery has emerged as a complementary intervention with profound metabolic effects. Bariatric surgery, as a complementary intervention to pharmacological treatments, plays a pivotal role in modifying the gut hormonal milieu, thereby enhancing the glucose regulatory and weight loss effects of semaglutide [142]. Furthermore, Roux-en-Y gastric bypass surgery exhibits particular potential in this domain, especially in influencing glucose excretion in the small intestine. A recent study revealed that Roux-en-Y gastric bypass surgery enhances glucose excretion in the small intestine by activating the AREG/EGFR/mTOR/AKT/GLUT1 signaling pathway, thereby improving glycemic levels [143]. Research shows that any type of weight loss surgery outperforms nonsurgical interventions in improving outcomes related to weight reduction and macrovascular complications in T2DM patients [144]. However, drastic weight fluctuations may also increase cardiovascular risk [145]. Studies indicate that in T2DM patients, weight changes exceeding 5% in one direction within two years are associated with increased risks of major cardiovascular events, emphasizing the importance of maintaining weight stability [145].

### Pharmacological prevention

#### Glycemic management

Glycemic management in T2DM patients has traditionally centered on glycated hemoglobin (HbA1c) levels as the principal parameter for assessing glycemic control and predicting the risk of macrovascular complications [114, 146–148]. If lifestyle modifications do not sufficiently lower HbA1c levels to below 7%, as recommended for patients with early-stage diabetes and without complications based on UKPDS observations and recent meta-analysis, the initiation of antidiabetic medication is justified [4, 88]. However, recent insights have led to a paradigm shift in our understanding of hyperglycemia. It is now recognized that effective glycemic management encompasses not only the achievement and maintenance of optimal HbA1c levels but also the reduction of postprandial hyperglycemia, the minimization of glycemic variability, and the maximization of time spent within the near-normoglycemic range, as these conditions are independently associated with macrovascular complications [106, 107]. Achieving these multifaceted goals while avoiding hypoglycemia is paramount to effective diabetes management. Contemporary therapeutic approaches, including the use of metformin, glucagon-like peptide-1 receptor agonists (GLP-1RAs), sodium-glucose cotransporter-2 (SGLT-2) inhibitors, and the innovative tirzepatide, have been proven effective at mitigating or halting the progression of atherosclerosis and reducing macrovascular complications [4, 103, 128, 149–153]. Importantly, this protective effect cannot be solely attributed to the hypoglycemic action of these agents, as other antidiabetic agents with potent hypoglycemic effects have not demonstrated similar benefits. Together with modern technologies such as intermittently scanned glucose monitoring and continuous glucose monitoring, these new drug therapies herald a more holistic approach to managing T2DM [107].

Metformin exerts multiple protective effects that are beneficial for vascular health, impacting smooth muscle cells, endothelial function, the lipid profile, and inflammation [154]. Experimental studies indicate that metformin promotes reverse cholesterol transport and reduces plaque cholesterol accumulation [155]. Additionally, improvements in endothelial function and reductions in inflammatory responses contribute to the atheroprotective effects of metformin [155]. Activation of AMP-activated protein kinase and alterations in the gut microbiota appear to be integral to the mechanism of action of metformin [156, 157].

SGLT-2 inhibitors have shown promising trends in the secondary prevention of macrovascular complications in diagnosed T2DM patients in multiple clinical trials and basic experiments [5, 52, 158, 159]. These inhibitors function by reducing intracellular sodium ions, thereby preventing oxidative stress and myocardial cell death [159]. Moreover, they activate various pathways related to cellular homeostasis, including autophagy pathways, thereby reducing the activation of inflammasomes and myocardial cell dysfunction [159, 160]. Interestingly, these agents induce a hibernation-like metabolic adaptation characterized by physiological adjustments that resist energy and fluid shortages [161]. By promoting glycosuria and alleviating hyperglycemia, SGLT-2 inhibitors not only address the inherent metabolic dysregulation of diabetes but also modulate inflammatory pathways, leading to vascular complications [162]. Specifically, the inhibition of SGLT2 has been shown to attenuate the activation of the key mediator NLRP3 inflammasome, thereby reducing the secretion of proinflammatory cytokines, such as interleukin-2β [162]. This modulation of inflammatory responses, coupled with metabolic regulation, underscores the dual protective mechanisms offered by SGLT2 inhibitors in the prevention of macrovascular complications in diabetes patients. Large-scale observational studies conducted in clinical settings have also confirmed the short-term clinical efficacy of SGLT2 inhibitors in reducing cardiovascular events in patients with T2DM [163]. Following the reported efficacy of SGLT-2 inhibitors in reducing macrovascular complications in patients with T2DM, a recent study emphasized their early use. This research, utilizing data from the AMD Annals, revealed that initiating SGLT-2 inhibitor therapy soon after T2DM diagnosis significantly reduces long-term cardiovascular risk, challenging the traditional delay in pharmacological intervention [164]. These results not only reinforce the role of SGLT-2 inhibitors in reducing cardiovascular events but also highlight their potential in altering the course of T2DM when SGLT-2 inhibitors are introduced early, suggesting a paradigm shift toward proactive and preemptive treatment strategies in diabetes care.

In clinical trials, the extended use and higher dosages of GLP-1RAs have demonstrated efficacy in preventing ischemic stroke and reducing the incidence of adverse cardiovascular events in Asian individuals with T2DM who do not have CVD [165, 166]. Intriguingly, foundational studies have demonstrated that GLP-1RAs have antiproliferative effects on VSMCs and ECs; mitigate oxidative stress, inflammation, and apoptosis; and promote the generation of NO and microvascular recruitment [152]. In clinical applications, patients receiving a triple therapy regimen that included GLP-1RAs have exhibited the lowest risk of significant cardiovascular adverse events [167]. SAR441255, a synthetic peptide agonist based on the exendin-1 sequence, simultaneously activates GLP-1, GIP, and GCG receptors and has shown superior weight loss and glycemic control effects in animal models and healthy subjects, with good tolerability [168].

Although both classes of drugs can reduce the risk of major adverse cardiovascular events, myocardial infarction, and cardiovascular mortality, a critical question arises: which medication should be prescribed for which patients in the pursuit of precision medicine for diabetes [5]? Generally, sodium glucose cotransporter 2 (SGLT-2) inhibitors have been shown in clinical trials to significantly reduce rates of hospitalization for heart failure, cardiovascular mortality, and renal outcomes. In contrast, clinical trials of GLP-1RAs have demonstrated significant improvements in major adverse cardiovascular events, including myocardial infarction and stroke [5, 149]. Furthermore, GLP-1RAs offer additional benefits by effectively reducing HbA1c levels, postprandial hyperglycemia, glucose variability, and hypoglycemia [107]. Similarly, SGLT2 inhibitors, whose glucose-lowering action does not rely on insulin, are also effective at lowering postprandial hyperglycemia and glucose variability while reducing the risk of hypoglycemia and increasing the time in range [107]. Additionally, a study on the clinical characteristics and prescription preferences for these two classes of drugs revealed that preferences for empagliflozin among SGLT2 inhibitors have evolved with changes in drug labeling and guidelines, whereas for GLP-1RAs, other factors, such as cost or ease of use, may have led to a preference for dulaglutide over liraglutide [169]. Globally, SGLT2 inhibitors appear to have an advantage in reducing diabetes complications and meeting cost objectives, particularly in low- and middle-income countries [170]. In summary, the choice of treatment for diabetes should be individualized based on the patient's risk profile and characteristics.

A crucial aspect of T2DM management is achieving a balance between effective glycemic control and minimizing the risk of hypoglycemia. Current guidelines advocate a lenient approach to glycemic control to minimize the risk of hypoglycemia [171]. Recent advances in novel therapeutic agents not only show immense potential in the secondary prevention of ASCVD in patients with T2DM but also offer promising strategies for this purpose. Along with a tolerable safety profile, tirzepatide, a novel dual agonist of glucose-dependent insulinotropic polypeptide and GLP-1RAs, has demonstrated unprecedented improvements in both glycemic control and weight reduction across a range of clinical trials [153]. As evidenced by research, tirzepatide not only has the potential to effectively reduce HbA1c levels to as low as 5.7% without causing significant hypoglycemia but also contributes to substantial weight loss and overall metabolic health improvement in individuals with T2DM [172].

#### Lipid management

The adoption of interventions of different intensities based on the risk of ASCVD is the core strategy for the prevention and treatment of dyslipidemia, and the overall ASCVD risk assessment is the basis for treatment decisions related to dyslipidemia [173]. Among the commonly used lipid markers, the one that is causally related to the risk of ASCVD development and is the primary clinical therapeutic target is low-density lipoprotein cholesterol (LDL-C) [173]. For diabetic patients aged 40 to 75 years who do not have established ASCVD, it is advisable to aim for an LDL-C target less than 70 mg/dL (< 1.8 mmol/L) or even as low as 55 mg/dL, especially for those who present additional risk factors for ASCVD174. In patients aged > 75 years with diabetes, setting LDL-C goals necessitates a balanced consideration of the potential benefits and risks, aligning with the individual's specific health context [174].

Statins and other lipid-lowering medications have been established as the most effective pharmacological agents for the prevention and treatment of ASCVD. Clinical studies have shown a significant increase in the use of these medications, particularly statins, which have been found to reduce the two-year risk of a first ischemic stroke by approximately half in T2DM patients without prior ASCVD [175]. Comprehensive management of lipid abnormalities, including those of LDL-C and lipoproteins, is considered central to mitigating cardiovascular risk factors and reducing cardiovascular risk [5, 176]. A meta-analysis encompassing 42 randomized controlled trials revealed that high- and moderate-intensity rosuvastatin, high-intensity simvastatin, and atorvastatin significantly lowered non-HDL-C levels and the risk of major cardiovascular events compared to placebo [177]. However, there are divergent opinions concerning the widespread use of statins. A retrospective study emphasized that the use of statins is associated with an increased likelihood of diabetes exacerbation, including elevated risks of initiating insulin therapy, deterioration of glycemic control, and an increase in the prescription of antidiabetic medications [178]. The underlying mechanism may be attributed to the demonstrated effect of statins in increasing insulin resistance, which is linked to various complications of diabetes [178]. Therefore, when assessing the overall risk–benefit profile of statin use in patients with diabetes, long-term impacts on quality of life and treatment burden should be considered.

The landmark REDUCE-IT trial suggested a paradigm shift in managing patients with high triglyceride (TG) levels, particularly those at high risk, such as patients with T2DM, metabolic syndrome, or obesity [179]. In addition to using statins to lower LDL-C levels, eicosapentaenoic acid should be administered to target elevated TG levels, offering a more comprehensive strategy for reducing ASCVD risk [179]. Recent guidelines from the European Medicines Agency propose that icosapent ethyl/eicosapentaenoic acid (IPE/EPA) may have significant preventive and therapeutic implications for macrovascular complications in T2DM patients, especially in its highly purified and stable form, IPE [180]. Research indicates that IPE/EPA can effectively lower elevated triglyceride levels and improve lipid profiles, potentially exerting a stabilizing influence on atherosclerotic plaques [180]. Additionally, IPE/EPA possesses anti-inflammatory properties, endothelial protective effects, and oxidative stress mitigation, among other benefits, all of which are advantageous for patients with T2DM experiencing macrovascular complications [180].

Annual lipid concentration testing is essential for effective lipid monitoring [176]. Globally, medications for lowering lipid and LDL-C levels in T2DM patients are far from optimal and present substantial untapped potential [181]. Moreover, significant global disparities exist in treatment regimens [182]. The World Health Organization's Global Diabetes Compact aims to increase the proportion of patients with diabetes (aged 40 years or older) receiving statin therapy to at least 60% and to control the blood pressure of 80% of patients to below 140/90 mmHg [183]. Particularly in low- and middle-income countries, efforts should focus on expanding the initiation and titration of antihypertensive and statin therapies to reduce diabetes complications [184].

#### Blood pressure management

Blood pressure management is an established strategy for preventing both microvascular and macrovascular complications in patients with T2DM. Mendelian randomization studies have substantiated the significance of BMI and systolic blood pressure in the progression of vascular complications in T2DM patients [130]. However, there is a divergence in the recommended blood pressure targets across medical societies. In the Action to Control Cardiovascular Risk in Diabetes (ACCORD) Blood Pressure Trial (ACCORD BP), it was not confirmed that a systolic blood pressure of < 120 mmHg in diabetic patients would lead to a reduction in cardiovascular event rates [185]. Therefore, it is recommended that hypertensive diabetic patients follow a more relaxed target, such as a blood pressure target of < 130/80 mmHg, as advocated by the ACC/AHA, AACE/ACE, and ESC/EASD. Moreover, the ADA proposes a risk-based treatment approach in which blood pressure is determined based on the 10-year risk of ASCVD [186]. Further research has suggested that standard blood pressure measurements may not adequately account for the extent and duration of exposure to elevated blood pressure over time. The cumulative systolic blood pressure load may offer a more accurate prediction of major cardiovascular events in T2DM patients than in healthy individuals [187].

Effective blood pressure reduction, achievable through lifestyle modifications and antihypertensive medications, can mitigate the risk of macrovascular complications in T2DM patients [186, 188–191]. Medical societies universally endorse the use of ACE inhibitors, ARBs, calcium channel blockers, and diuretics to achieve these targets [186]. When combination therapy is needed, these societies recommend the concurrent use of either an ACE inhibitor or ARB with a calcium channel blocker or diuretic while discouraging the simultaneous use of ACE inhibitors and ARBs [186].

Comprehensive management of glycemic levels (measured via hemoglobin A1C), blood pressure, and non-HDL cholesterol levels, along with smoking cessation, are considered core components of a preventive strategy [192, 193]. Objective monitoring and tailored interventions are crucial for enhancing patient adherence. Clinical trials employing biochemical urine tests to assess T2DM patient compliance with oral antidiabetic drugs, antihypertensive medications, and statins have shown that nonadherent patients exhibit significantly higher rates of both microvascular and macrovascular complications and are less likely to achieve treatment goals [194]. State-level prevalence surveys indicate that only approximately one-fourth of adults with diabetes in the United States meet comprehensive management targets, underscoring the need for public health sectors to further amplify efforts to disseminate intensified management objectives for T2DM patients to reduce diabetes-related complications [193].

#### Antithrombotic therapy

The increased vascular risk in patients with diabetes is attributed to early and aggressive atherosclerotic disease, compounded by an enhanced thrombogenic milieu [5, 62]. This prothrombotic environment is characterized by increased platelet activity and alterations in both the quantity and quality of coagulation factors, leading to impaired fibrinolysis [62]. d-dimer, a fibrin degradation product released into the bloodstream during fibrinolysis, serves as a reliable biomarker for thrombosis and is associated with the severity of PAD, multivascular disease, and adverse CAD events [2].

Current therapeutic strategies for improving vascular outcomes in patients with diabetes primarily focus on prompt revascularization of acute atherosclerotic obstructions, coupled with early initiation of antithrombotic therapy, typically achieved through platelet function inhibition [62]. Antiplatelet agents, including monotherapies (e.g., aspirin or P2Y12 inhibitors) and dual antiplatelet therapy (DAPT) (e.g., concurrent use of aspirin and P2Y12 inhibitors), hold indispensable value as secondary preventive pharmacological agents for patients with diabetes [52]. Post hoc analyses have revealed that, in T2DM patients with stable CAD and no history of myocardial infarction or stroke, ticagrelor plus aspirin yields a net clinical benefit compared to placebo plus aspirin, encompassing endpoints such as all-cause mortality, myocardial infarction, and stroke [195]. Moreover, research by Cebo et al. demonstrated that the chemokine receptor CXCR7 modulates thrombo-inflammatory platelet function via the production of "antithrombotic" lipid species, suggesting a potential new strategy for targeted thrombo-inflammation [196]. Combined antiplatelet and anticoagulant therapy, particularly the coadministration of aspirin and low-dose rivaroxaban (a factor Xa inhibitor), has been proven effective in reducing the risk of CAD or PAD in stable T2DM patients [2, 52, 197, 198]. In a targeted trial involving 19,909 patients receiving nonvitamin K antagonist oral anticoagulants and 10,300 patients receiving warfarin for atrial fibrillation and diabetes, it was found that those treated with nonvitamin K antagonist oral anticoagulants had a significantly reduced risk of major vascular complications compared to those treated with warfarin [199]. However, the need for regular monitoring of warfarin and other vitamin K antagonists, along with the elevated bleeding risk when warfarin is used in combination with antiplatelet therapy, has hindered their widespread adoption [62].

It is particularly important to discuss in depth the benefits and risks of bleeding tendencies when employing dual antiplatelet therapy or antiplatelet plus anticoagulant treatment strategies in individuals who meet the criteria for T2DM patients. A shared decision-making approach should be adopted to determine a treatment plan tailored to the individual. The latest guidelines and clinical trials have shown that dual antiplatelet therapy or antiplatelet plus anticoagulant treatment strategies are associated with a higher incidence of major bleeding events, including intracranial hemorrhage [174, 195]. However, in terms of secondary prevention for patients with diabetes, intensifying antithrombotic treatment strategies beyond just using aspirin is recommended for patients who can accept the risk of bleeding, as demonstrated by insights from the COMPASS Trial [197].

#### Molecular targeted therapies

Traditional pharmacological interventions, such as antihyperglycemic, lipid-lowering, antihypertensive, and antiplatelet therapies, have demonstrated efficacy in mitigating macrovascular complications in diabetes patients to some extent. However, these approaches often target systemic metabolic pathways and may not fully address the underlying pathophysiological mechanisms responsible for vascular complications. Advances in molecular biology and genetics have ushered in targeted therapies aimed at specific molecules or signaling pathways implicated in the pathogenesis of diabetes-associated macrovascular complications. These targeted interventions offer the potential for more precise treatment with potentially fewer side effects.

Various strategies for targeted therapies for diabetic macrovascular complications include inflammation, immune modulation, antioxidation and endothelial function protection, metabolic regulation, cellular senescence and lifestyle interventions. In patients with T2DM, despite having LDL-C controlled at optimal levels (< 70 mg/dL), persistent elevation of high-sensitivity C-reactive protein above 2 mg/L poses a residual inflammatory risk, emerging as a new risk factor for cardiovascular events in high-risk atherosclerotic patients [200]. The RESCUE trial explored the role of ziltivekimab, a targeted IL-6 inhibitor, in reducing inflammation and thrombotic markers in patients at high cardiovascular risk [201]. The study showed that ziltivekimab significantly lowers hs-CRP levels, particularly in type 2 diabetes patients with chronic kidney disease and elevated hs-CRP levels. These findings emphasize the importance of addressing inflammatory pathways in diabetes management, in addition to controlling blood sugar and cholesterol levels, and offer new therapeutic strategies to reduce cardiovascular complications. Furthermore, in the context of inflammation and immune modulation, the inhibition of the NLRP3 inflammasome and the use of specific small-molecule NLRP3 inhibitors, such as MCC950, have shown promise in reducing inflammation and improving vascular function in animal models [79]. These studies suggest that targeting NLRP3-mediated inflammation has substantial therapeutic potential by slowing the progression of atherosclerosis and enhancing plaque stability. Second, in terms of antioxidation and endothelial function protection, acidic fibroblast growth factor (aFGF) and melatonin have been shown to have significant effects [16, 102]. aFGF improves endothelial function by inhibiting mitochondrial oxidative stress, while melatonin achieves this by preventing S-nitrosylation of GNAI2 [16, 102]. In metabolic regulation, MGO, a precursor of AGEs, has been identified as a key factor in the pathogenesis of vascular complications associated with diabetes [7, 71, 72]. MGO contributes to the nonenzymatic glycation of proteins and DNA, leading to structural and functional alterations in organs and tissues [7, 71, 72]. Interestingly, the induction of glyoxalase 1 has been shown to detoxify MGO, suggesting a potential therapeutic strategy to alleviate these vascular complications [7, 71, 72]. Thus, targeting both MGO and glyoxalase 1 could provide a comprehensive approach to managing vascular complications in diabetes patients.

Additionally, gut hormones and the nuclear receptor farnesoid X receptor (FXR) play key roles in glucose homeostasis. Inhibition of FXR or its downstream target gene sphingomyelin phosphodiesterase 3 significantly reduces atherosclerosis induced by a high-cholesterol diet [202, 203]. The gastrointestinal system plays a pivotal role in the regulation of glucose homeostasis, with specific gut hormones and FXR serving as key regulators of this system [202, 203]. FXR, in particular, is expressed in the gut and is implicated in various metabolic disorders, including obesity, fatty liver disease, and T2DM. Targeting FXR or its downstream gene sphingomyelin phosphodiesterase 3 has been shown to significantly attenuate atherosclerosis induced by a high-cholesterol diet [202, 203]. Innovations in this field have also emerged from research targeting cellular aging and lifestyle modifications. For instance, modulation of the gut microbiota, particularly through the use of *Odoribacter laneus* as a probiotic, has been shown to lower circulating levels of succinic acid, improve glucose tolerance, and ameliorate inflammatory conditions [68]. Additionally, mitochondrion-targeted interventions, such as tamoxifen, have yielded promising results in improving glucose tolerance, reducing body weight, and decreasing hepatic lipid accumulation [204].

Notably, these molecular targeted therapies are often integrated with other treatment modalities to achieve more comprehensive and effective therapeutic outcomes. For example, the first-line pharmacotherapy metformin optimizes therapeutic effects by modulating the gut microbiome [205]. Heat shock proteins, particularly HSP70, are molecular targets for insulin resistance [206]. This multifaceted, multilayered therapeutic strategy reflects a significant trend in current diabetes treatment research and provides a broad scope for future studies on personalized treatment, combination therapies, drug repurposing, clinical trials, real-world studies, and novel drug delivery systems. In summary, multimodal pharmacological strategies, including lipid-lowering, antiplatelet, antihypertensive, and antidiabetic agents, in addition to novel drugs targeting specific pathological mechanisms, offer effective secondary prevention of macrovascular complications in patients with T2DM. These interventions should include diabetes self-management education, smoking cessation, appropriate sleep duration, and psychosocial care to achieve precision prevention and individualized targets [5, 207].

### Multidisciplinary risk assessment and management plans

In the realm of secondary prevention for diabetes and its macrovascular complications, multidisciplinary risk assessment and management plans have emerged as pivotal therapeutic approaches endorsed by the American Diabetes Association, the European Society of Cardiology and the European Association for the Study of Diabetes [208]. Given the chronic nature of diabetes, which necessitates lifelong management, patients are advised to engage continuously with the healthcare system, ideally at the primary care level [208]. Through team-based care coordination, clinical care and diabetes self-management support are integrated, involving primary care providers, other healthcare professionals, and administrative staff [208]. Specifically, healthcare practitioners stratify patients based on their risk factors and offer tailored management recommendations. For those categorized as high risk or with poor HbA1c control, additional consultations and services, including patient empowerment programs, patient support call centers, and smoking cessation counseling, are provided by RAMP-DM nurses and physicians. Patients undergo RAMP-DM assessments every 1–3 years and receive regular physician follow-up, similar to non-RAMP-DM participants [209, 210]. Randomized clinical trials have demonstrated that this multidisciplinary, team-based approach involving chronic care models and individualized management plans serves as an effective adjunct to standard primary care for mitigating or delaying the onset or progression of macrovascular and microvascular complications or mortality in diabetes patients [208].

Moreover, the success of this program is not solely dependent on the expertise of physicians, nurses, and nutritionists; it also emphasizes active community participation and comprehensive support from the healthcare system [211]. Community involvement is crucial for tailoring preventive measures to the actual needs and cultural backgrounds of patients, thereby enhancing the acceptability and feasibility of these interventions [211]. Additionally, systemic support, including funding and training for multidisciplinary teams, enables this multilayered, multidisciplinary approach to comprehensively address the complex needs of diabetes patients in terms of quality of life, disease management, and prognosis[211].

**Table 1:** Clinical trials have shown that intervention measures reduce the risk of major vascular complications in patients with T2DM

Intervention	Method	Primary outcome	Risk reduction ratio	References
Lifestyle interventions	Medical nutrition therapy	All-cause mortality, CVD mortality events, stroke	7–32%	[63, 121, 123]
	Weight reduction	Cardiometabolic diseases, cardiac diastolic function	0.84–2.45times	[131, 133]
	Increased physical activity			
	Including different types of physical activity (dynamic aerobic exercise (126/248 trials), dynamic resistance exercise (25/248 trials), and combined aerobic and resistance exercise (58/248 trials))	All-cause mortality: median 6 months:	15 fewer per 1.000 (22 fewer to 6 fewer);	[135]
		Cardiovascular mortality: median 12 months	24 fewer per 1.000 (35 fewer to 11 fewer);	
		Myocardial Infarction: median 12 months	8 fewer per 1.000 (16 fewer to 3 more);	
		Stroke: median 3.6 months:	2 fewer per 1.000 (10 fewer to 9 more)	
	Including different types of physical activity (total physical activity, leisure-time physical activity, moderate-to-vigorous physical activity, and walking)	Total CVD incidence	A high compared with low level of physical activity was associated with a 16%	[136]
		Coronary heart disease incidence	A high compared with low level of physical activity was associated with a 16%	
		Cerebrovascular events	A high compared with low level of physical activity was associated with a 26%	
		heart failure incidence	A high compared with low level of physical activity was associated with a 24%	
Pharmacological prevention	Glycemic management	Macrovascular event (coronary heart disease, heart failure, stroke, and peripheral arterial disease)	0%-39%	[146, 147]
		macrovascular events (Myocardial Infarction, Congestive Heart Failure, stroke, angina, and revascularization)	8–48%	[148]
	Metformin	Heart Failure	18%	[154]
	GLP-1RAs	Stroke, MACE, all-cause and cardi138ovascular mortality	12–72%	[149, 151, 158, 165]
	SGLT-2 inhibitors	All-cause and cardiovascular mortality, serious heart failure events	16–40%	[158, 159]
	Combined medication			
	Dual therapy(metformin + DPP-4i/sulfonylurea/SGLT2i/GLP-1-RA/basal insulin)	MACE	36–79%	[167]
	Triple therapy ( metformin + DPP-4i + sulfonylurea/SGLT2i + GLP-1-RA/GLP-1-RA 1 basal insulin)		38–83%	
	Lipid management			
	Statins	Nonfatal myocardial infarction	43%	[177]
	IPE/EPA	ASCVD	4.8–23.3%	[179, 180]
	Blood pressure management	MACE, all-cause mortality, cardiovascular death	13–34% (Each 1-SD) SBP	[187, 189–192]
	Thrombosis treatment ( Antiplatelet drugs (Aspirin/P2Y12 receptor antagonists/Other antithrombotic approaches) with or without anticoagulant drugs (rivaroxaban))	all-cause death, stroke, myocardial infarction, MACE	8.7–39%	[62, 195, 197, 198]

MACE: major adverse cardiovascular event; ASCVD: atherosclerotic cardiovascular disease

## Experimental models for studying macrovascular complications of type 2 diabetes

### Animal models

Selecting appropriate models is crucial for secondary prevention research for T2DM. Rodent models, notably mouse and rat models, are commonly used due to their physiological similarities to humans [27]. Genetically engineered models, such as ob/ob and db/db mice and the atherosclerosis models ApoE−/− and LDLR−/−, are invaluable for studying T2DM-related atherosclerosis. These models have been used to elucidate the cellular and molecular mechanisms driving macrovascular complications and serve as platforms for therapeutic evaluation [27, 79, 212]. Additionally, microbiota inoculation and bone marrow transplantation offer novel avenues for understanding the roles of the gut microbiome and hematopoietic cells in disease progression [13, 68].

### Cellular models

Cellular models, including human aortic smooth muscle cells and bone marrow-derived macrophages, are indispensable for T2DM research [79, 213, 214]. These models complement in vivo studies but face challenges in replicating human physiology due to the complexity of vascular structures [215].

Organoid technology has become increasingly important, especially for secondary prevention of T2DM [216]. Recent advances include human capillary vascular organoids, human islet-like organoids and engineered islets from stem cells, which offer promising avenues for diabetes research [217–219]. As research advances, we expect the creation of in vitro systems capable of simulating specific organ ECs. These future systems may also include peripheral cells to better replicate the vascular environment. A recent study demonstrated the ability to reset mature human ECs, enabling them to form stable, functional vascular networks in three-dimensional matrices [220]. Future in vitro systems may incorporate features to simulate blood flow and perfusion, enhancing the accuracy of vascular environment simulation [216].

While rodent models are extensively used, their physiological differences from humans limit their translational potential [27]. The comprehensive application of various models and methods allows for a more holistic understanding of the complex mechanisms of macrovascular complications in diabetes [27]. Due to space constraints in this review, a comprehensive summary table (Table 2) is provided for a foundational understanding of these experiments.

**Table 2:** Comparison of experimental models in secondary prevention research for macrovascular complications of type 2 diabetes

Experimental model	Application scenario	Advantages	Limitations	References
Rodent Models (Mice and Rats)	Atherosclerosis, hyperglycemia, hyperinsulinemia, dyslipidemia, gut microbiome, hematopoietic cells	High reliability, physiological relevance, cost-effective study designs, advanced genetic manipulation	Physiological differences from humans, experimental complexity	[13, 27, 68]
Cellular Models (Human Aortic Smooth Muscle Cells, Bone Marrow-Derived Macrophages, Human Umbilical Vein Endothelial Cells, Human Aortic Endothelial Cells)	Vascular smooth muscle cells, macrophages, endothelial cells	Direct approach, cost efficiency	In vitro environment limitations	[79, 213–215]
Organoid Models	Metabolic disease symptoms, drug screening, evaluation	Flexibility, human specificity	Cost, technical complexity	[216, 218, 219]
3D Culture Matrices and Microfluidic Chambers (e.g., R-VECs)	Endothelial cell research, vascular network reconstruction	Human environment mimicry, vascular generation, repair utility	Cost, technical complexity	[220]

## Comprehensive experimental analysis and technological translation of atherosclerosis associated with macrovascular complications of diabetes

### Basic research

Recent advances in modern imaging technologies have significantly expanded our ability to visualize and characterize atherosclerotic plaques at the cellular and molecular levels. These technologies offer intricate views of plaque complexity and encompass five key aspects: plaque visualization, quantification, cellular composition, cell-specific markers or transcriptomes and endothelial dysfunction. Due to space constraints in this review, a comprehensive summary table (Table 3) is provided for a foundational understanding of these experiments.

**Table 3:** Summary of the experimental analysis and research output for models of diabetes-associated atherosclerosis

Analysis	Technique	Research output	References
Plaque visualization	Noninvasive nanoprobe (OPN Ab/Ti3C2/ICG)	Distinguishing vulnerable plaques and achieving noninvasive specific in vivo imaging	[221]
	Peroxynitrite (ONOO) activatable probe (CNP2-B)	High selectivity and signal-to-noise ratio imaging	[222]
	Intravascular fluorescence lifetime	Biochemical changes	[223]
	Fluorescent-colorimetric probe (Pro-P1)	Simultaneously, used for visualizing lipoprotein oxidation and plaque imaging	[224]
	Lipid-activatable fluorescent probe (CN-N2)	Intraoperative imaging	[225]
	Adipo-clear (three-dimensional imaging)	Morphology (volume, geometry, acellular component, surface, and spatial position within the BCA)	[226]
Plaque quantification	Intravascular fluorescence lifetime	Biochemical changes	[223]
	3-Dimensional volumetric ultrasound	Volume	[227]
Plaque cellular composition	High-resolution multispectral fluorescence lifetime imaging microscopy	Principal compositions of coronary arteries (tunica media, tunica adventitia, elastic laminas, smooth muscle cell-enriched fibrous plaque, lipid-rich core, and foamy macrophages)	[237]
	Miller elastin/Van Gieson staining, Picrosirius red staining, hematoxylin and eosin staining	Elastin, fibrillar collagen, necrotic core	[238]
	BODIPY, ORO staining, immunofluorescent analysis, flow cytometry	VSMC-derived foam cell formation	[239]
	Immunofluorescence staining, oil red O staining	Vascular smooth muscle cell c-Fos, foam cell	[240]
	Enzyme‐linked immunosorbent assay (ELISA), hematoxylin and eosin staining (H&E), oil red O staining	Macrophages, nuclear factors, vascular cells	[228]
Cell-specific markers or transcriptomes	Single-cell RNA sequencing, single-cell ATAC sequencing	Transcriptomic and epigenomic characteristics	[229]
	Single-cell RNA sequencing	Gene expression of bone marrow-derived monocytes/macrophages	[230]
	Single-cell RNA sequencing	A novel cell state during smooth muscle cell phenotypic switching	[234]
	Flow cytometry	Plaque- and immunological phenotyping	[231]
	Immunohistochemistry, proximity extension assays, and Helios cytometry by time of flight	Major macrophage associated genes and transcription factors	[235]
Endothelial dysfunction	Vessel myography	Endothelium-dependent function, endothelium-independent function	[236]

While traditional clinical approaches have been limited in their capacity to identify vulnerable atherosclerotic plaques at the molecular level, recent innovations are bridging this diagnostic gap. Notably, an advanced nanoprobe (OPN Ab/Ti3 C2/ICG) with augmented optoacoustic capabilities enables direct and noninvasive in vivo visualization of such plaques [221]. Building on this, researchers are intensifying efforts to enhance the specificity and sensitivity of imaging technologies. A recent study employed an "AND" logic gate to develop an activatable fluorescent probe with a high signal-to-noise ratio [222]. When accurately administered in a murine atherosclerosis model, this probe demonstrated superior selectivity and signal fidelity in in vivo imaging [222]. Advances in fluorescence imaging are offering invaluable insights into key phenomena related to plaque progression [223]. A newly developed probe, Pro-P1, which is triggered by oxidized low-density lipoprotein, facilitates the direct observation of lipid oxidation and aggregation within live cells [224]. In the surgical context, the ability to visualize the full extent of atherosclerotic plaques during procedures has a significant impact on therapeutic outcomes. Research indicates that lipid-activated probes for visible fluorescence bioimaging can be used to precisely delineate plaques with diameters smaller than 0.5 mm [225]. In addition to examining cellular and molecular dimensions, advances in imaging technologies are providing a more comprehensive understanding of tissue architecture. A novel technique, "adipo-clear", has been developed for whole-tissue visualization and immunolabeling [226]. By utilizing light-sheet microscopy, three-dimensional images and reconstructions are generated, revealing intricate interactions between tissue structures and components related to embryonic brown fat development [226]. In a controlled ex vivo setting, researchers utilized state-of-the-art three-dimensional vascular ultrasound for precise volumetric assessment of plaques, demonstrating significant concordance with histological gold standards [227].

In addition to advances in imaging technologies, a myriad of studies have elucidated the utility of histological staining, immunohistochemistry, and immunofluorescence in delineating the cellular composition of atherosclerotic plaques. These methodologies have been particularly instrumental in investigating key molecules and mechanisms implicated in the formation of VSMCs and foam cells, such as the roles of c-Fos, P2RY12 receptors, and galectin-3 [228–231]. Complementing these approaches, a separate line of research employed rapid aortic freezing in murine models to facilitate RNA and protein extraction, followed by quantitative reverse transcription PCR and Western blot analyses to further characterize proteins and pathways associated with atherosclerosis [228].

To gain a more nuanced understanding of the transcriptomic cellular landscape of atherosclerotic plaques, research teams have employed an integrative approach leveraging cutting-edge technologies, such as lineage tracing, single-cell RNA sequencing, human genomics, and flow cytometry. These studies have particularly focused on previously overlooked immune cell subpopulations and their roles in disease progression [229–231]. Furthermore, single-cell RNA sequencing has revealed significant heterogeneity in immune cells within human atherosclerotic lesions [232]. The design, implementation, and interpretation of these single-cell data have undergone comprehensive review and guidance [233]. Building on this foundation, the integration of cell-specific fate mapping, single-cell genomics, and human genetics has provided novel perspectives on the complexities of SMC biology. These investigations have unveiled new cellular states involved in SMC phenotypic transitions and potential therapeutic targets in both murine and human atherosclerosis [234]. Multiple techniques, including RNA sequencing, immunohistochemistry, and time-of-flight cytometry, have further underscored the pivotal role of the transcription factor interferon regulatory factor-5 in human atherosclerosis. Specifically, these studies have demonstrated a close association between the expression of this transcription factor and macrophage phenotypes, plaque inflammation, and vulnerability to rupture [235]. Ex vivo vasomotor kinetics serve as another critical technique for assessing endothelial viability and function, which are key indicators of early-stage atherosclerosis associated with diabetes. This methodology has aided researchers in evaluating the impact of endothelial-specific HDAC2 overexpression on endothelium-dependent vasorelaxation and atherosclerotic lesion formation [236].

### Clinical applications

#### Nanomedicine

The risk of complications in patients with diabetes is exacerbated by oxidative stress and macrovascular damage induced by hyperglycemia. Fortunately, recent advances in nanomedicine offer novel avenues for both the diagnosis and treatment of diabetes. Early diagnosis can guide interventions, such as lifestyle modifications or pharmacotherapy, aimed at mitigating glycemic abnormalities and preventing associated complications. Biosensors are increasingly utilized to identify key diabetes biomarkers, such as glucose in human breath, HbA1c levels, insulin, and acetone gas [241]. Innovative approaches, including color-changing contact lenses and paper-based assays for glucose and acetone, show promise for real-time and noninvasive diabetes monitoring, potentially enhancing patient convenience and adherence to regular monitoring regimens [241]. To prevent macrovascular complications, symptom control is imperative and is typically achieved through blood glucose monitoring and timely medication. There is an urgent need for noninvasive monitoring and drug delivery strategies to minimize associated discomfort. In this regard, nanomaterials have demonstrated multifaceted applications, serving as drug delivery vehicles that display insulin-mimetic activities, possess antioxidative properties, and target the aggregation of human islet amyloid polypeptides [241]. For instance, researchers have developed a multifunctional, minimally invasive transdermal metabolic monitoring system based on silicon nanowire field-effect transistors [242]. This system is embedded in microneedles and allows real-time monitoring of metabolites in subcutaneous interstitial fluid. Preliminary murine studies have validated its ability to monitor glucose simultaneously and for insulin delivery [242]. This cost-effective, reliable wearable device holds promise as a powerful adjunct for both patients and researchers, enabling direct and synchronous biomarker monitoring and transdermal drug delivery.

#### Biomechanics and bioengineering applications

Stringent glycemic control is paramount in diabetes management to mitigate the onset of late-stage complications, with self-monitoring of blood glucose being the initial recommendation. However, adherence to daily self-monitoring is suboptimal in more than half of the population with diabetes owing to the painful, invasive nature of blood sampling, the risk of infection and the costs associated with nonreusable devices. To circumvent these limitations, numerous studies have explored noninvasive technologies, such as integrated microneedle biosensors fabricated via 3D printing, for continuous diabetes monitoring, demonstrating accurate subcutaneous glucose level measurements [243].

A recent study investigated the application of bioelectronic noses guided by artificial neural processing for the diagnosis of diabetes and its cardiomyopathic complications. Although the study did not specifically address macrovascular complications, its academic significance lies in its comprehensive approach, employing advanced M13 bacteriophage electronic noses and multilevel biological feature analysis, from cellular to murine models, for precise diagnosis and classification of various types of diabetes and its complications [244]. Techniques such as wire myography and shear stress analysis offer valuable tools for assessing vascular function and physiological responses [79, 245]. These technologies not only elucidate the underlying disease mechanisms but also provide insights into potential therapeutic targets and preventive strategies. Noninvasive cardiac output monitoring is an advanced technique for real-time measurement of cardiac output and other hemodynamic parameters to assess vascular stiffness. However, this technique primarily focuses on systemic hemodynamics and offers limited evaluation of local cardiac vascular structure and function. Speckle-tracking echocardiography has emerged as an advanced cardiac imaging technology to fill this gap, enabling precise assessment of microstructural myocardial changes in T2DM patients who may be elusive in conventional echocardiographic examinations [246]. Tissue engineering and 3D printing of bioartificial pancreases have also demonstrated potential applications in diabetes treatment [247].

#### Applications in medical imaging

Advances in a myriad of imaging modalities have significantly augmented the identification of atherosclerotic plaques. Among these methods, magnetic resonance imaging (MRI), computed tomography (CT), positron emission tomography (PET), single-photon emission computed tomography (SPECT), ultrasound (US), optical imaging (OI), optical coherence tomography (OCT), and photoacoustic imaging (PAI) are most prevalently employed for the diagnostic evaluation of patients at potential risk. Studies corroborate that cardiovascular imaging serves as an invaluable tool in guiding therapeutic interventions aimed at mitigating complications. For instance, the utilization of computed tomography or contrast ultrasound has been associated with a reduction in overall cardiovascular risk and improvements in individual risk factors, such as cholesterol levels and systolic blood pressure [248].

According to expert consensus, CT serves as the diagnostic modality of choice for excluding obstructive stenosis in patients with a moderate pretest probability of CAD. It offers quantitative assessment of coronary plaque dimensions, composition, and location as well as associated cardiovascular risk, particularly the volume of noncalcified and low-density plaques [249–251]. MRI, however, facilitates the visualization of coronary plaques and may serve as a radiation-free alternative for coronary angiography in experienced centers [250]. Its high resolution and absence of ionizing radiation render it pivotal for evaluating plaque composition and stability. PET holds the greatest potential for quantifying inflammation within coronary plaques, although SPECT currently has limited utility in the clinical imaging of coronary stenosis and atherosclerosis [250]. Intravascular ultrasound and optical coherence tomography are the most critical invasive imaging techniques for identifying high-risk rupture plaques [248, 250, 252]. PAI combines the advantages of optical and ultrasound imaging, providing high-resolution structural, hemodynamic, and biochemical information. Specifically, PAI offers unique benefits in the early diagnosis of atherosclerosis, endothelial function assessment, and real-time monitoring of microvascular perfusion and oxygenation states, as well as drug delivery and therapeutic efficacy [253].

However, these technologies are not without limitations. High-end equipment such as MRI and PET are often cost-prohibitive in low-income or resource-limited settings. Ionizing radiation-based imaging modalities, such as CT, PET, and SPECT, may restrict their application in certain populations, including pregnant women and children. Additionally, the limited penetration depth of optical imaging and OCT renders them unsuitable for assessing deeper tissues. Therefore, the selection of an appropriate imaging modality requires comprehensive consideration of factors such as resolution, detection depth, cost, acceptability, and specific patient needs. Multimodal imaging strategies that integrate these strengths and mitigate the limitations of various imaging modalities may represent the future direction of clinical applications. The recommendations in this consensus statement will assist clinicians in selecting the most appropriate imaging modality based on specific clinical scenarios, individual patient characteristics, and the availability of each imaging modality [250]. To facilitate a foundational understanding of the key attributes of these medical imaging technologies, a comprehensive summary table is provided (see Table 4).

**Table 4:** Comparison of medical imaging technologies for the identification of atherosclerotic plaques in the secondary prevention of macrovascular complications in patients with type 2 diabetes

Imaging technology	Resolution	Detection depth	Characteristics for plaque identification	Advantages	Disadvantages
MRI	High	High	Plaque Composition and Stability	No Radiation, High Contrast	Expensive, Time-Consuming
CT	Medium	High	Plaque Calcification and Location	Fast, High Resolution	Radiation Risk
PET	Low	High	Plaque Activity and Inflammation	High Sensitivity	Expensive, Radioactive Drugs
SPECT	Low	High	Plaque Activity and Inflammation	High Sensitivity	Radiation Risk, Low Resolution
US	Medium	Medium	Plaque Morphology and Blood Flow	Non-Invasive, Real-Time	Limited Detection Depth
OI	High	Low	Plaque Surface Features	High Resolution	Limited Detection Depth
OCT	High	Low	Plaque Microstructure	High Resolution, No Radiation	Limited Detection Depth, Expensive Equipment
PAI	High	Medium	Plaque Composition and Blood Flow	High Resolution, Multimodal	Technically Complex, Expensive Equipment

## Discussion

### Principal findings and contributions

In this comprehensive review, we have undertaken an exhaustive and nuanced examination of the mechanisms underlying the secondary prevention of macrovascular complications in patients with T2DM. Our work serves to bridge a notable gap in the literature, offering clinicians and researchers an integrated framework for reference.

Initially, we investigated the pathophysiological mechanisms, including dysregulated glucose metabolism, chronic inflammation, alterations in hemorheology, and HIFs. Through meticulous analysis, we elucidated how these mechanisms synergistically contribute to the onset and progression of macrovascular complications, particularly atherosclerosis. Subsequently, we highlighted the pivotal role of protective mechanisms in secondary prevention, an aspect hitherto underrepresented in prior research. We elaborated on various preventive strategies, including biomarker monitoring and management, pharmacological interventions, and lifestyle modifications, thereby offering invaluable guidance for clinical practice. With respect to experimental methodologies, we discussed the utility and significance of animal and cellular models, which facilitate a more profound understanding of pathophysiological mechanisms and the evaluation of preventive strategy efficacy.

In summary, this review furnishes a holistic perspective on the secondary prevention of macrovascular complications in T2DM patients, advancing both academic research and clinical practice. Nevertheless, we also acknowledge the limitations of the current research and propose directions for future inquiry, including personalized treatment approaches, multidisciplinary research, and considerations of global and regional disparities, to further deepen our understanding and refine preventive and therapeutic strategies.

### Limitations of the study

While this review offers a comprehensive and nuanced exploration of the mechanisms underlying the secondary prevention of macrovascular complications in T2DM patients, spanning pathophysiological mechanisms, protective factors, preventive strategies, and experimental methodologies, it is imperative to acknowledge certain non-negligible limitations. First, given the heterogeneity and complexity of related research, our review does not encapsulate all conceivable preventive strategies and mechanisms, particularly those in nascent stages of investigation. Second, although we assessed individual preventive measures, our understanding of the synergistic and cumulative effects of multiple interventions is relatively limited. This is primarily attributable to the preponderance of existing studies focusing on singular preventive strategies, with a dearth of long-term follow-up studies examining the integrated application of multiple strategies.

Furthermore, while our literature selection prioritized studies published post-2020 to capture the most recent advances in the field, this criterion may inadvertently overlook seminal research from earlier periods. Finally, the precise identification of T2DM patients at elevated risk as well as the effective translation of basic research findings into clinical practice continue to pose significant challenges in this area. In summary, although this review furnishes a comprehensive perspective on the secondary prevention of macrovascular complications in T2DM patients, further research is needed to fill these knowledge and practice gaps, thereby offering valuable guidance for future research directions.

### Recommendations and directions for future research

T2DM is a multifactorial, heterogeneous disorder, and its progression involves intricate interactions among genetic, environmental, and lifestyle factors [6]. To enhance the prevention of macrovascular complications, future research should focus on the following pivotal issues and directions.

Personalized Treatment Strategies: T2DM is a complex, multifactorial disease composed of genetic, environmental, and lifestyle factors [254]. This heterogeneity is manifested in diverse clinical outcomes and responses to treatment, necessitating a shift toward personalized treatment strategies [254]. Recognizing the various pathophysiological pathways leading to hyperglycemia, including dysfunction of β-cells and altered incretin responses, underscores the opportunity to tailor treatments more effectively based on the underlying pathophysiology. Precision medicine, leveraging big data analytics, offers unprecedented opportunities to customize care for individual patients, including drug treatment plans and lifestyle interventions [255]. For instance, a nuanced understanding of EC diversity can inform the development of targeted therapies to meet the specific vascular characteristics of individual patients [9]. The differential expression of metabolic genes in cardiac/muscle ECs compared to other tissues highlights the potential for organ-specific therapeutic interventions [9]. Clinical evidence underscores the differential impact of therapeutic drugs such as SGLT-2 inhibitors and GLP-1RAs on macrovascular complication outcomes, heralding a new era of phenotype-driven clinical care [5, 149]. However, integrating genetic data into routine clinical management remains an aspiration yet to be realized on a broad scale, with the potential to enhance therapeutic efficacy by tailoring treatments to genetic susceptibility [256]. To alleviate the burden of macrovascular complications, a detailed understanding of risk factors, from BMI to lipid profiles, is essential. The development of biomarker-based risk stratification tools holds promise for facilitating early detection and intervention, potentially transforming the diabetes management landscape. As these tools are refined, their validation across different populations is crucial to ensure global applicability and to address the nuances of diabetes risk and its inherent complications.

Multidisciplinary Research Approaches: Given the complexity of T2DM and its macrovascular complications, future studies should adopt multidisciplinary methodologies. This encompasses, but is not limited to, integrative considerations of biology, pharmacology, epidemiology, health economics, and psychosocial factors. Revisions to existing guidelines to bolster multidisciplinary usage and the implementation of patient and clinician education may encourage the adoption of these medications, potentially improving long-term health outcomes for T2DM patients [257].

Global and Regional Variations: Research indicates that T2DM and its complications manifest differently across various regions and populations, even exhibiting multilevel disparities within the same ethnic group [211]. Addressing these issues necessitates a comprehensive research and practice framework. Funding bodies should support projects employing community engagement and multidisciplinary research approaches.

Economic Disparities: Economic disparities exert a profound influence on the management of T2DM. The cost of pharmacological treatments and socioeconomic factors are pivotal in determining access to care, with insurance coverage posing additional barriers [257]. Studies, such as those of the Whitehall II cohort, highlight the disproportionate burden of T2DM in socioeconomically marginalized groups, emphasizing the need to address broader socioeconomic, sex, racial, and ethnic disparities [258]. In response to these challenges, global initiatives such as the WHO Global Diabetes Compact advocate for comprehensive strategies that transcend individual-level interventions. A central focus of these strategies is insulin access, a critical component of the diabetes care ecosystem. By ensuring equitable insulin availability, particularly in low- and middle-income countries, these initiatives aim to reduce the disparity in diabetes outcomes [259]. Additionally, enhancing community healthcare capabilities is crucial [260]. Empowering trusted community members to bridge the gap between underserved communities and healthcare services can significantly improve diabetes care networks [259]. Such community-centric approaches, coupled with global efforts to address systemic healthcare inequities, are essential for reducing the burden of T2DM. These measures collectively aim to mitigate macrovascular complications and improve the quality of life for individuals living with diabetes, thereby addressing the global challenge of diabetes-related health disparities.

In summary, future research should strive to develop more personalized treatment strategies, delve deeper into disease mechanisms, employ multidisciplinary approaches, and effectively translate research findings into clinical practice. This will contribute to improving the efficacy of preventive measures for macrovascular complications in patients with T2DM, alleviating the disease burden on patients, and fostering further advances in this field.

## Data Availability

Data sharing is not applicable for this article, as no new data were created or analyzed in this study.

## Abbreviations

T2DM: Type 2 diabetes mellitus
CAD: Coronary artery disease
PAD: Peripheral artery disease
ECs: Endothelial cells
25-HC: 25-Hydroxycholesterol
LDL: Low-density lipoprotein
AGEs: Advanced glycation end products
RAGE: Receptor for advanced glycation end product
HIF: Hypoxia-inducible factor
ER: Endoplasmic reticulum
UPR: Unfolded protein response
PERK: Protein kinase RNA-like endoplasmic reticulum kinase
ROS: Reactive oxygen species
BMI: Body mass index
NO: Nitric oxide
eNOS: Endothelial nitric oxide synthase
BCAAs: Branched-chain amino acids
MGO: Methylglyoxal
EVs: Extracellular vesicles
TNF-α: Tumor necrosis factor-α
CVDs: Cardiovascular diseases
IL-6: Interleukin-6
VSMCs: Vascular smooth muscle cells
EPCs: Endothelial progenitor cells
NT-proBNP: N-terminal pro-brain natriuretic peptide
PPCs: Peripheral vascular progenitor cells
BAT: Brown adipose tissue
MG53: Mitsugumin 53
DAPT: Dual antiplatelet therapy
aFGF: Acidic fibroblast growth factor
Cyr61: Cysteine-rich angiogenic inducer 61
HbA1c: Glycated hemoglobin
SGLT-2: Sodium-glucose cotransporter-2
GLP-1RAs: Glucagon-like peptide-1 receptor agonists
ASCVDs: Atherosclerotic cardiovascular diseases
LDL-C: Lipoprotein cholesterol
TG: Triglyceride
IPE/EPA: Icosapent ethyl/eicosapentaenoic acid
FXR: Farnesoid X receptor
MRI: Magnetic resonance imaging
CT: Computed tomography
PET: Positron emission tomography
SPECT: Single-photon emission computed tomography
US: Ultrasound
OI: Optical imaging
OCT: Optical coherence tomography
PAI: Photoacoustic imaging
